# *In situ* generation of dendritic cell vaccines in 3D printing scaffolds for cancer post-surgical therapy

**DOI:** 10.1093/nsr/nwag037

**Published:** 2026-01-20

**Authors:** Lefan Chen, Yangtao Xu, Xiao Hu, Yuanwei Pan, Peng She, Xiaoyuan Chen, Qiyu Peng, Qi Li, Lang Rao

**Affiliations:** Department of Urology, The First Affiliated Hospital of Zhengzhou University, Zhengzhou 450052, China; Institute of Chemical Biology, Shenzhen Bay Laboratory, Shenzhen 518132, China; Institute of Chemical Biology, Shenzhen Bay Laboratory, Shenzhen 518132, China; Cancer Center, Renmin Hospital of Wuhan University, Wuhan 430060, China; Institute of Chemical Biology, Shenzhen Bay Laboratory, Shenzhen 518132, China; Institute of Chemical Biology, Shenzhen Bay Laboratory, Shenzhen 518132, China; Department of Diagnostic Radiology, Yong Loo Lin School of Medicine, National University of Singapore, Singapore 119074, Singapore; Department of Orthopedics, The Seventh Affiliated Hospital of Sun Yat-Sen University, Shenzhen 518000, China; Department of Diagnostic Radiology, Yong Loo Lin School of Medicine, National University of Singapore, Singapore 119074, Singapore; Nanomedicine Translational Research Program, Yong Loo Lin School of Medicine, National University of Singapore, Singapore 117597, Singapore; Institute of Molecular and Cell Biology, Agency for Science, Technology, and Research (A*STAR), Singapore 138673, Singapore; Institute of Biomedical Engineering, Shenzhen Bay Laboratory, Shenzhen 518132, China; Department of Urology, The First Affiliated Hospital of Zhengzhou University, Zhengzhou 450052, China; Institute of Chemical Biology, Shenzhen Bay Laboratory, Shenzhen 518132, China

**Keywords:** DC vaccine, 3D printing, tumor lysate, irradiation, cancer immunotherapy

## Abstract

Dendritic cell (DC) vaccines have shown great promise in cancer management, while complex *ex vivo* cell culture limits their clinical applications. Here, we report an *in situ*-generated scaffold DC vaccine, comprising a simple 3D-printed gelatin methacryloyl, bone marrow mononuclear cells (BM-MNCs), tumor lysates and stimulating factors. Stimulating factors in this vaccine effectively initiated *in situ* differentiation of BM-MNCs into DCs, while personalized tumor lysates stimulated the maturation of differentiated DCs and enhanced their lymph node migration efficiency, thereby synergistically strengthening their antitumor effects. Moreover, the 3D hydrogel scaffold serves as an *in situ* cell culture matrix, promoting the long-term viability of encapsulated cells and providing a conducive environment for BM-MNC differentiation and DC maturation within the surgical bed. In a prostate cancer post-surgical mouse model, the vaccine significantly inhibited tumor growth, suppressed tumor metastasis and extended median survival of animals to 55 days. This *in situ*-generated DC vaccine bypasses *ex vivo* cell culture, offering a simple, safe and robust strategy to elicit antitumor immunity for cancer treatment.

## Teaser

This work reports a 3D-printed scaffold vaccine, allowing in situ differentiation and maturation of BM-MNCs into dendritic cells toward robust antitumor immunity without ex vivo culture.

## INTRODUCTION

Surgical resection is the primary therapeutic modality for solid tumors [1]. However, despite significant advancements in surgical techniques, post-operative residual occult micrometastases and circulating tumor cells pose a substantial risk of tumor recurrence and metastasis [1–3]. Immune checkpoint blockade (ICB) treatment that reactivates T cells within tumors has shown great potential in reducing the risk of recurrence and metastasis following surgery [4–6]. However, sustained clinical responses are elicited by systemic ICB therapy in fewer than 20% of patients with immunogenic tumors [2,7]. Furthermore, its efficacy is markedly limited in malignancies featuring low tumor mutational burden, such as prostate cancer [8]. Additionally, side effects including autoimmune diseases secondary to ICB remain a concern [9,10]. Given the inherent limitations of systemic ICB therapy, there is a pressing need for alternative or complementary immunotherapeutic strategies to induce tumor-specific T cells.

An alternative approach to induce robust tumor-specific T cells relies on antigen presentation by dendritic cells (DCs) [11–13]. DC vaccines, which constitute a pivotal strategy in cancer immunotherapy, have demonstrated significant antitumor potential in both preclinical and clinical investigations [14–16]. However, conventional DC vaccines necessitate multi-step *ex vivo* procedures, including monocyte isolation, induced differentiation and antigen loading processes [14]. This complex process, due to its inherent personalized manufacturing complexity, results in a protracted manufacturing timeline of at least 2–3 weeks and substantial costs, thereby significantly hindering broad clinical translation [17–19]. Therefore, it is necessary to develop a DC vaccine preparation strategy that establishes an *in vivo* environment for DC vaccine generation, to bypass the complex and time-consuming *ex vivo* cell culture process.

Gelatin methacryloyl (GelMA) is a widely used biomaterial for 3D cell culture and tissue engineering due to its biocompatibility, tunable mechanics and cell adhesion sites [20–22]. However, bulk hydrogels suffer from limited mass transport, restricting nutrient diffusion and waste removal, impairing cell viability in thick constructs [23,24]. As a form of augmented manufacturing, 3D printing enables fabrication of porous architectures with enhanced permeability, significantly improving nutrient and waste exchange and enhancing cell survival and delivery [23,25,26], while light-curing-based approaches provide rapid, low-shear GelMA crosslinking that preserves immune cell viability and uniformity, making them well suited for the fabrication of cell-laden scaffolds [27,28]. In addition to delivery, autologous tumor lysates (TL) as antigens can provide a wide range of tumor antigen stimulation, which is critical for DC vaccines as well [11,29,30]. Critically, irradiation-induced damage-associated molecular pattern (DAMP)-rich TLs (DTL) promote DC maturation and enhance antigen presentation via pattern-recognition receptor activation, and thus have the potential to further promote the efficiency of DC vaccines [12,31].

**Figure 1.**
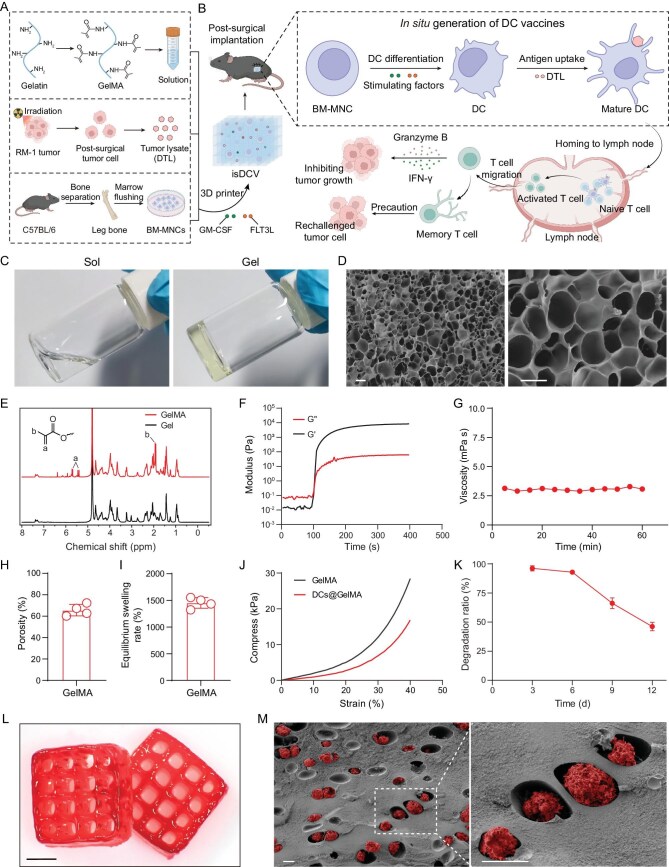
Schematic and characterization of isDCV. (A) Schematic showing the isDCV was 3D bioprinted with bioink loaded with BM-MNCs, DTL, FLT3L and GM-CSF (figure made in BioRender). (B) Following implantation into the surgical tumor cavity, the isDCV facilitates the differentiation of BM-MNCs into DCs. These DCs subsequently take up and present tumor-associated antigens, which stimulates their maturation. The mature DCs then migrate to dLNs where they prime naive CD8^+^ T cells. Finally, the activated CD8^+^ T cells traffic back to the tumor site, mediating specific cytotoxic lysis of tumor cells and establishing a long-lasting antigen-specific memory (figure made in BioRender). (C) GelMA’s sol–gel transition in response to 405 nm flashlight illumination. (D) SEM of GelMA hydrogel. Scale bars, 10 μm. (E) ^1^H NMR spectra of GelMA hydrogel. (F) Dynamic time-sweep rheological analysis of GelMA hydrogel exposed to a dose of 30 mW cm^−2^ of 405 nm laser. (G) The viscosity changes of the GelMA hydrogel. (H) The porosity of freeze-dried GelMA hydrogel. (I) The equilibrium swelling rate of the GelMA hydrogel. (J) The compressive stress-strain curves of the GelMA and DCs@GelMA. (K) Characteristic of scaffold after extraction *ex vivo*. (L) The manufacturing and stability of the 3D porous scaffold. Scale bar, 5 mm. (M) isDCV SEM imaging. Scale bars, 10 μm. All data are expressed as mean ± SD (H, I: *n* = 4; K: *n* = 3).

Here, we report an *in situ*-generated DC vaccine (isDCV) strategy wherein a GelMA hydrogel co-encapsulates undifferentiated bone marrow mononuclear cells (BM-MNCs), granulocyte-macrophage colony-stimulating factor (GM-CSF), Flt-3 ligand (FLT3L), and personalized DTL (Fig. 1A). Implanted into murine tumor resection cavities during surgery, isDCV enables *in situ* differentiation of BM-MNCs into DCs. Upon stimulation with personalized DTL, DCs mature and subsequently exhibit enhanced homing capacity of draining lymph nodes (dLNs), thereby promoting a sustained antitumor immune response [23]. In prostate cancer mouse models, the isDCV significantly enhanced the infiltration of CD8^+^ cells into tumor tissues and markedly suppressed tumor growth, suppressed tumor metastasis and extended median survival (Fig. 1B). Furthermore, we use 3D printing technology to enable personalized vaccine development for site-specific implantation following tumor resection. This *in situ* DC vaccine strategy, bypassing *ex vivo* cell culture, marks a new era in cancer vaccination.

## RESULTS

### GelMA solution as a DC bioink

GelMA hydrogels have been widely used as a delivery platform for drugs and cells, owing to their favorable biocompatibility, cost-effectiveness and photo cross-linkable nature [24,32]. Successful synthesis was confirmed by a sol–gel transition under irradiation (405 nm light source) (Fig. 1C). Following photocuring and lyophilization, morphological characterization via scanning electron microscopy (SEM) revealed a highly porous network with a range of pore dimensions (Fig. 1D). This interconnected porosity is expected to facilitate nutrient diffusion and waste metabolite transport, potentially supporting cellular functions such as the differentiation of BM-MNCs into DCs. Conjugation efficiency of methacryloyl (MA) groups onto gelatin (Gel) was validated by the emergence of characteristic ‘C=C’ peaks in ^1^H nuclear magnetic resonance (NMR) spectroscopy (Fig. 1E). Furthermore, dynamic rheological time-sweep tests demonstrated GelMA could be light-cured in less than 20 s after being exposed to a 405 nm laser (30 mW cm^−2^ irradiation dose) (Fig. 1F), indicating that GelMA hydrogels demonstrate a rapid photocuring ability. Additionally, the GelMA’s viscosity changed little over time (Fig. 1G). Porosimetric assessment of the lyophilized hydrogels indicated porosity exceeding 60% (Fig. 1H), which would enhance the efficiency of nutrient uptake and waste elimination within the cells. The hydrogel displayed an equilibrium swelling ratio of >1400% (Fig. 1I, Fig. S1), which is indicative of its capacity to adapt to aqueous environments and effectively retain liquid-phase nutrients. Subsequent mechanical characterization revealed a decrease in compressive strength post-cell incorporation (Fig. 1J), potentially complicating subsequent printing.

3D bioprinting has emerged as a transformative strategy in biomedical applications, owing to its ability to fabricate constructs containing viable cells, known as bioinks [33]. This technology enables spatially controlled deposition of bioinks based on digital anatomical models via computer guidance, positioning it as a highly promising modality within tissue engineering [34,35]. In this study, we investigated the 3D printability of a bioink formulation comprising a physical admixture of GelMA solution and BM-MNCs. Capitalizing on the photocurable nature of GelMA, hydrogel constructs were fabricated using projection micro stereolithography (PμSL) printing. The bioink permitted high-fidelity printing of diverse, architecturally complex scaffolds corresponding to computer-aided design models (Fig. S2A). Subsequent to 3 days of culture in a cell culture medium, these constructs maintained favorable morphological integrity and sustained cellular viability (Fig. S2B), demonstrating the hydrogel’s excellent cell loading capacity. *In vivo* degradation analysis revealed a mass loss of approximately 50% over a 12-day period (Fig. 1K and Fig. S2D), which ensured adequate time for the *in vivo* generation of DC vaccines. Consistently, SEM imaging before and after *in vivo* degradation confirmed substantial microstructural changes in the hydrogel (Fig. S3). Bioprinted 3D porous scaffolds exhibited high structural fidelity and defined porosity (Fig. 1L and Fig. S2C). More importantly, cross-sectional SEM imaging and confocal z-stack analysis further confirmed the homogeneous distribution of BM-MNCs within the hydrogel (Fig. 1M and Fig. S4), indicating excellent biocompatibility.

### Stimulatory effects of DTL on BMDCs

**Figure 2.**
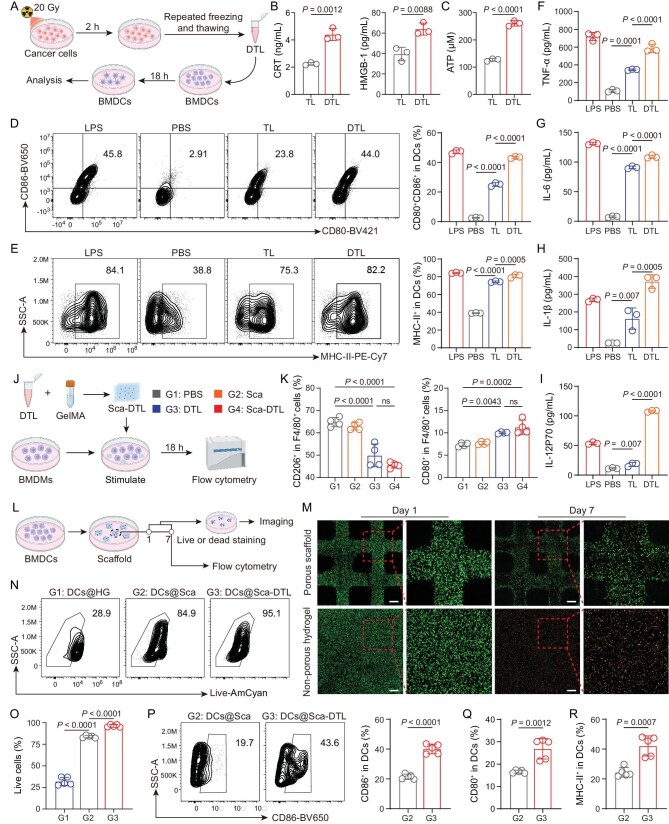
DTL as immunomodulators for BMDCs *ex vivo*. (A) Schematic illustration of preparation of DTL and culture process of BMDCs and DTL (figure made in BioRender). (B and C) Concentration of CRT, HMGB-1 (B) and ATP (C) between TL and DTL of RM-1 cells. (D) Representative flow cytometry analysis and quantitation of the percentage of DC maturity in BMDCs incubated with PBS, LPS, TL and DTL. (E) Representative flow cytometry analysis and quantitation of the proportion of MHC-II^+^ in DCs. (F–I) The concentrations of cytokines TNF-α (F), IL-6 (G), IL-1β (H) and IL-12p70 (I) secreted by BMDCs after stimulation. (J) Schematic illustration of the culture process of BMDMs and Sca-DTL (figure made in BioRender). (K) Quantitation of the percentage of M2-like macrophages (CD206^+^) and M1-like macrophages (CD80^+^) on F4/80^+^CD11b^+^ cells incubated with PBS, Sca, DTL and Sca-DTL. (L) The schematic illustration of experimental design (figure made in BioRender). (M) Live and dead staining of DCs in porous scaffold and non-porous hydrogel. Scale bars, 500 μm. (N and O) Representative flow cytometry analysis (N) and quantitation of the percentage (O) of cell viability in BMDCs loaded in non-porous hydrogel (HG), Sca and Sca-DTL. (P) Representative flow cytometry analysis and quantitation of the proportion of CD86^+^ in DCs. (Q and R) Proportion of CD80^+^ (Q) and MHC-II^+^ (R) in DCs. All data are expressed as mean ± SD (B–I: *n* = 3; K: *n* = 4; O–R: *n* = 5). Statistical significance was calculated via ordinary one-way analysis of variance (ANOVA) with a Tukey’s test (D, E, F, G, H, I, K, O) or unpaired two-tailed *t*-test (B, C, P, Q, R). ns, no significance.

Irradiation induces immunogenic cell death in tumor cells, triggering the release of DAMPs [11]. Recognition and binding of these DAMPs stimulate the activation and maturation of DCs, enabling them to phagocytose dying tumor cells, process tumor antigens and present them to T cells [36,37]. To generate DTL, tumor cells were irradiated with X-rays and then cultured for an additional 2 h. The cells were subsequently harvested and subjected to successive freeze–thaw cycles, sonication and centrifugation (Fig. 2A). Enzyme-linked immunosorbent assay (ELISA) detection revealed significantly elevated levels of calreticulin (CRT) and high-mobility group box 1 (HMGB1) in DTL compared to untreated TL (Fig. 2B). Immunofluorescence analysis confirmed a marked upregulation of CRT surface exposure on RM-1 cells at 2 h post-irradiation (Fig. S5A). Furthermore, quantification using an ATP detection kit demonstrated increased ATP levels in DTL relative to TL (Fig. 2C). These results indicate that irradiation significantly increases the levels of CRT, HMGB1 and ATP in DTL.

Bone marrow-derived DCs (BMDCs) were treated with phosphate-buffered saline (PBS), lipopolysaccharide (LPS), TL or DTL. Flow cytometric analysis revealed that DTL treatment resulted in a robust, concentration-dependent increase in the expression of co-stimulatory molecules CD80 and CD86 on BMDCs after 18 hours (Fig. 2D and Fig. S5B). Additionally, DTL exposure markedly enhanced the surface expression of major histocompatibility complex class II (MHC-II) molecules (Fig. 2E). These findings collectively indicate that DTL effectively promote BMDC maturation and their antigen-presenting function. Supernatants collected from DCs treated with the different agents were analyzed for cytokine secretion. As anticipated, results correlated with the flow cytometry data: DTL treatment significantly augmented the production of tumor necrosis factor-alpha (TNF-α), interleukin-6 (IL-6), interleukin-1 beta (IL-1β) and interleukin-12p70 (IL-12p70) compared to TL treatment (Fig. 2F–I), providing further evidence for DTL’ potent capacity to induce DC maturation. Furthermore, DTL at a concentration of 200 μg/mL exerted no discernible cytotoxicity on BMDCs (Fig. S5C).

Beyond activating DCs, DAMPs possess the ability to modulate the tumor microenvironment (TME) [38,39]. In this study, bone marrow-derived macrophages (BMDMs) were treated with PBS, 3D scaffold alone (Sca), DTL or the DTL-loaded 3D scaffold (Sca-DTL) (Fig. 2J). Flow cytometric analysis performed after 18 h of incubation revealed that DTL, both alone and in the Sca-DTL formulation, significantly reduced expression of the M2 marker CD206 while increasing expression of the M1-associated marker CD80 (Fig. 2K), indicating that DTL induced a shift from the M2 to the M1 phenotype in BMDMs. Crucially, this repolarizing effect was not impaired by incorporation within the hydrogel scaffold. These results demonstrate that DTL effectively induce M2-to-M1 macrophage repolarization, highlighting their potential to remodel the immunosuppressive TME.

### Survival and maturation of BMDCs in 3D scaffold

DCs exert their immunological function primarily through antigen presentation [40]. Therefore, the survival and antigen uptake capacity of DCs within hydrogels represent critical determinants for hydrogel-based DC vaccines. Immature BMDCs were mixed with GelMA solution and subsequently bioprinted into 3D porous scaffolds (Fig. 2L). BMDCs encapsulated within the 3D scaffolds exhibited significantly enhanced viability compared to those in non-porous hydrogels by Day 7 (Fig. 2M). Viability analysis revealed approximately 75% survival of DCs within the 3D porous scaffolds, contrasting sharply with only ∼12% survival in non-porous hydrogels (Fig. S6). These findings demonstrate that the 3D porous architecture sustains BMDC viability significantly better than non-porous hydrogels.

Furthermore, to evaluate maturation effects, BMDCs were encapsulated within different platforms: non-porous hydrogel; 3D porous scaffold without DTL (Sca); and 3D porous scaffold incorporating DTL (Sca-DTL). Following 7 days of culture in a cell culture medium, cells were retrieved from the hydrogels via lysis and subjected to flow cytometric analysis (Fig. 2L). The 3D porous scaffold significantly increased the proportion of viable BMDCs to ∼84% compared to ∼32% in the non-porous hydrogel control. Notably, incorporation of DTL within the scaffold further augmented BMDC survival to ∼96% (Fig. 2N and O), suggesting that 3D porous scaffolds possess superior BMDC culture capacity, and the DTL encapsulated within them also enhance BMDC survival. Additionally, BMDCs cultured within Sca-DTL exhibited significant upregulation of the co-stimulatory molecules CD80 and CD86 (Fig. 2P and Q, Fig. S7A). Concurrently, a marked increase in the expression of MHC-II molecules was observed in BMDCs cultured in Sca-DTL (Fig. 2R, Fig. S7B). These results indicate that Sca-DTL provides a supportive microenvironment, enabling efficient survival of BMDCs, while maintaining DTL-induced DC maturation and enhanced antigen-presenting capacity.

### *In situ* differentiation of BM-MNCs into DCs in 3D scaffold

**Figure 3.**
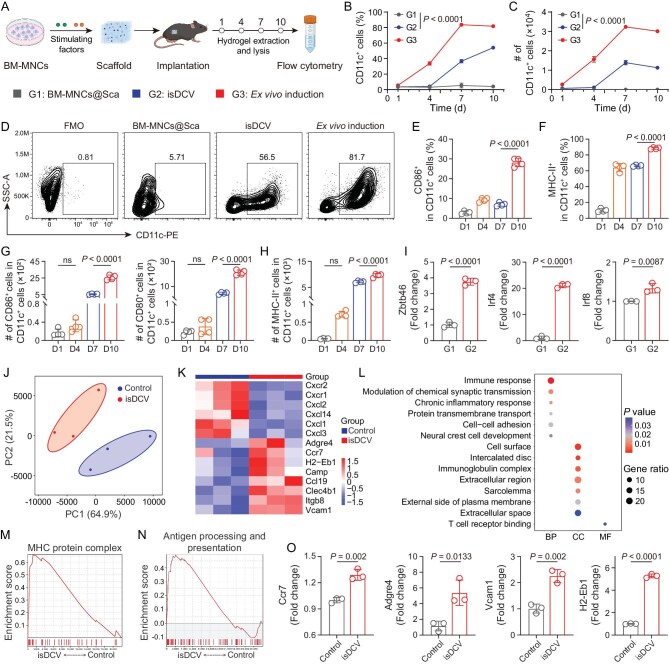
*In situ* differentiation of BM-MNCs and maturation of DCs. (A) Schematic illustration of the experimental design. BM-MNCs were loaded into hydrogels and implanted subcutaneously into mice. Flow cytometry analysis was performed on cells retrieved from the implanted hydrogels to assess *in situ* differentiation and maturation of DCs (figure made in BioRender). (B) Change of quantitation of the percentage of DCs in different groups over time. (C) Change of number of DCs in different groups over time. ‘#’ denotes number. (D) Representative flow cytometry analysis of DCs in live cells in BM-MNCs@Sca, isDCV and culture dish (*ex vivo* induction) on Day 10, with fluorescence minus one (FMO). (E) Quantitation of the proportion of CD86^+^ DCs in isDCV over time. (F) Quantitation of the proportion of MHC-II^+^ DCs in isDCV over time. (G and H) Quantitation of the number of CD86^+^ DCs, CD80^+^ DCs (G) and MHC-II^+^ DCs (H) in isDCV over time. ‘#’ denotes number. (I) RT-qPCR showing upregulated Zbtb46, Irf4 and Irf8 in the isDCV group on Day 10. (J) PCA scatter plot analysis of isDCV and control groups. (K) Heatmap of DEGs between isDCV groups and control groups. (L) The results of GO pathway enrichment analysis. (M) GSEA revealing activation of MHC protein complex signaling. (N) GSEA highlighting antigen processing and presentation pathway. (O) RT-qPCR showing upregulated Ccr7, Adgre4, Vcam1 and H2-Eb1 in isDCV group. All data are expressed as mean ± SD (B, C, E, F, G, H: *n* = 4; I, O: *n* = 3). Statistical significance was calculated via ordinary one-way ANOVA with a Tukey’s test (B, C, E, F, G, H) or unpaired two-tailed *t*-test (I, O). ns, no significance.

Next, to investigate the *in vivo* differentiation capacity of BM-MNCs into mature DCs within the implanted hydrogel microenvironment at the surgical tumor bed, 3D GelMA scaffolds loaded with BM-MNCs (BM-MNCs@Sca) or co-loaded with BM-MNCs, DTL, FLT3L and GM-CSF (isDCV) were implanted into the resected tumor cavity of C57BL/6 mice. Scaffolds were harvested on post-operative Days 1, 4, 7 and 10. Following hydrogel lysis, recovered cells were subjected to flow cytometric analysis to quantify DC differentiation. BM-MNCs induced to differentiate into BMDCs by traditional *ex vivo* culture served as positive control (Fig. 3A).

During the first 4 days post-implantation, no significant increase was observed in the proportion or absolute number of DCs within scaffolds from either BM-MNCs@Sca or isDCV groups compared to time zero levels. By Day 7, the isDCV group exhibited a pronounced differentiation shift towards the DC phenotype, achieving approximately 37% DCs as determined by flow cytometry. Notably, by Day 10, both the proportion and absolute number of DCs within the isDCV increased further, reaching approximately 57% DC differentiation (Fig. 3B–D and Fig. S8A). Concurrently, significant elevations in the proportions of CD86⁺ DCs and CD80⁺ DCs within isDCV were detected on Day 10 (Fig. 3E, Fig. S8B). Enumeration of cells within isDCV revealed significant temporal increases in the absolute numbers of CD86⁺ DCs and CD80⁺ DCs (Fig. 3G). Furthermore, both the proportion and the absolute number of MHC-II⁺ DCs displayed analogous upward trends over time (Fig. 3F and H). To further validate DC differentiation, quantitative real-time PCR (RT-qPCR) analysis was performed on cells encapsulated in scaffolds. The expression levels of DC marker genes, such as Zbtb46, Irf4 and Irf8, were significantly increased in the isDCV group compared with those in the BM-MNCs@Sca group (Fig. 3I) [41]. Collectively, the flow cytometry and RT-qPCR data demonstrate that BM-MNCs can undergo successful *in situ* differentiation into DCs directly within the hydrogel matrix *in vivo*. This approach effectively circumvents the need for complex personalized *ex vivo* generation procedures. Importantly, these *in situ*-generated DCs were functionally competent to stimulate adaptive immune responses, as demonstrated by enhanced TNF-α and interferon-gamma (IFN-γ) expression in T cells following co-culture (Fig. S9).

To further elucidate the underlying biological mechanism of isDCV, RNA sequencing analysis was performed on cells in untreated groups and isDCV groups. Based on the principal component analysis (PCA) scatter plot analysis, the data revealed distinct inter-group differences (Fig. 3J). Transcriptomic profiling of the isDCV group revealed marked transcriptomic remodeling (Fig. 3K). Heatmap analysis revealed that culturing in isDCV significantly upregulated the expression of key genes, including Ccr7, Vcam1, H2-Eb1 and Adgre4. This transcriptional upregulation suggests that isDCV induces critical immune functions, such as lymphocyte homing, antigen presentation and inflammatory responses [42–46]. Furthermore, these findings imply that DAMPs within isDCV activate inflammatory signaling pathways in DCs, thereby promoting their enhanced homing to dLNs for subsequent antigen presentation to T cells. To further elucidate the transcriptional programs regulated by isDCV, we performed Gene Ontology (GO) enrichment analysis and gene set enrichment analysis (GSEA) based on the bulk RNA-seq data. Consistently, GO enrichment analysis showed a strong association with immune response processes (Fig. 3L). GSEA pathway enrichment analysis revealed significant enrichment of the MHC protein complex pathway in isDCV-loaded DCs (Fig. 3M). Moreover, GSEA analysis further revealed significant enrichment of the antigen processing and presentation pathway in isDCV-loaded DCs (Fig. 3N). Finally, we validated the differentially expressed genes (DEGs) by RT-qPCR (Fig. 3O). These results provide further evidence supporting the DC-activating and lymph node-homing/promoting effects of isDCV.

### *In vivo* lymphatic homing of DCs in 3D scaffold

**Figure 4.**
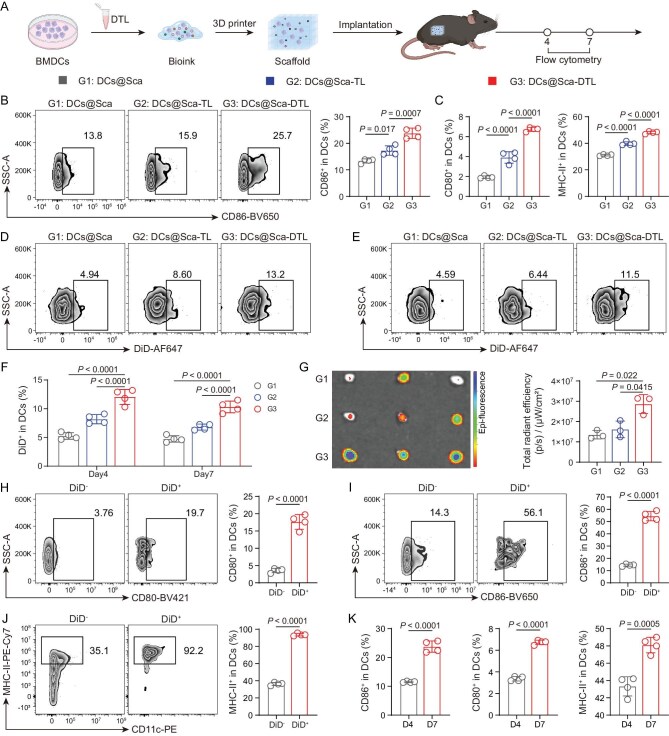
Lymphocyte migration capacity of DCs within porous scaffolds. (A) Schematic illustration of experimental design (figure made in BioRender). (B) Representative flow cytometry analysis and quantitation of the proportion of CD86^+^ DCs in the DCs of dLNs treated with DCs@3D scaffold (DCs@Sca), DCs@3D scaffold-TL (DCs@Sca-TL) and DCs@3D scaffold-DTL (DCs@Sca-DTL) on Day 7. (C) Quantitation of the proportion of CD80^+^ DCs and MHC-II^+^ DCs in the DCs of dLNs on Day 7. (D and E) Representative flow cytometry analysis of DiD^+^ in DCs on Days 4 (D) and 7 (E). (F) Quantitation of the percentage of DiD^+^ in DCs. (G) IVIS images and fluorescence intensities analysis of dLNs on Day 7. (H–J) Representative flow cytometry analysis and quantitation of the percentage of CD80^+^ (H), CD86^+^ (I) and MHC-II^+^ (J) in DiD^+^ DCs and DiD^−^ DCs in DCs@Sca-DTL on Day 7. (K) Quantitation of the percentage of CD86^+^ DCs, CD80^+^ DCs and MHC-II^+^ DCs in the DCs of dLNs on Day 4 and Day 7 in DCs@Sca-DTL. All data are expressed as mean ± SD (B, C, F, H, I, J, K: *n* = 4; G: *n* = 3). Statistical significance was calculated via ordinary one-way ANOVA with a Tukey’s test (B, C, F, G) or unpaired two-tailed *t*-test (H–K).

Lymphocytes surveil the body for antigens and initiate antigen-specific immune responses within dLNs [40]. Consequently, enhancing the homing capacity of DCs to dLNs is critical for effective DC-mediated immunity. We first characterized the release behavior of DTL from the hydrogel scaffold, which suggested that DTL exhibited a sustained and limited release profile rather than a rapid burst (Fig. S10). To evaluate the impact of DTL on DC homing within 3D scaffolds, we then subcutaneously implanted DCs@Sca, DCs@Sca-TL and DCs@Sca-DTL scaffolds into the surgical tumor beds of mice. On Day 7 post-implantation, dLNs were surgically excised for flow cytometric analysis (Fig. 4A). Notably, mice bearing DCs@Sca-DTL scaffolds exhibited a significantly increased proportion of CD86^+^ DCs within their dLNs compared to the DCs@Sca-TL group (Fig. 4B). Consistently, DCs@Sca-DTL scaffolds induced significantly higher proportions of CD80⁺ DCs and MHC-II⁺ DCs within the dLNs relative to the DCs@Sca-TL group (Fig. 4C, Fig. S11A and B). Additional *in vivo* experiments showed that DC maturation in dLNs was significantly higher in the DCs@Sca-DTL group than in the Sca-DTL group (Fig. S12), indicating that enhanced DC maturation in dLNs is mainly attributable to the migration of scaffold-derived DCs, with a limited contribution from DTL diffusion alone.

To verify that mature DCs detected in the dLNs originated from the implanted 3D scaffolds, DCs were pre-labeled with the lipophilic fluorescent tracer 1,1′-dioctadecyl-3,3,3′,3′-tetramethylindodicarbocyanine perchlorate (DiD) (Fig. S13). On Days 4 and 7 post-implantation, both the percentage and absolute number of DiD⁺ DCs within dLNs were significantly elevated in mice implanted with DCs@Sca-DTL scaffolds compared to those receiving DCs@Sca-TL and DCs@Sca scaffolds (Fig. 4D–F, Fig. S14). Furthermore, the proportion and number of DiD⁺ DCs in dLNs of DCs@Sca-DTL-treated mice remained stable between Days 4 and 7, indicating that DTL enhance DC homing to dLNs and the Sca-DTL scaffold sustains DC trafficking to dLNs over an extended duration (Fig. 4F, Fig. S14). Immunofluorescence and IVIS imaging of dLNs corroborated a markedly higher accumulation of DiD⁺ DCs within the dLNs of mice implanted with DCs@Sca-DTL scaffolds (Fig. 4G, Fig. S15). Given the potential detachment of lipophilic dyes, DC migration was further validated using intracellular carboxyfluorescein succinimidyl ester (CFSE) labeling. CFSE-labeled DCs were readily detected in dLNs at Days 4 and 7, with significantly higher frequencies and numbers in the DCs@Sca-DTL group compared with controls (Fig. S16A–D). IVIS imaging further confirmed enhanced DC homing to dLNs in the DCs@Sca-DTL group (Fig. S16E).

Additionally, DiD⁺ DCs displayed significantly higher expression levels of the maturation markers CD80, CD86 and MHC-II compared to DiD⁻ DCs (Fig. 4H–J, Fig. S11C–E), demonstrating that the exogenously implanted DCs homing to dLNs maintain a highly mature phenotype. Concurrently, the proportions of CD86⁺ DCs, CD80⁺ DCs and MHC-II⁺ DCs within the dLNs increased over time (Fig. 4K, Fig. S11F), suggesting that sustained DC homing to dLNs enhances dLNs’ antigen-processing and antigen-presentation capacity. Collectively, these results demonstrated that DTL potentiate the migration of DCs to dLNs, thereby enhancing antitumor immune efficacy.

### *In vivo* antitumor effects of isDCV

**Figure 5.**
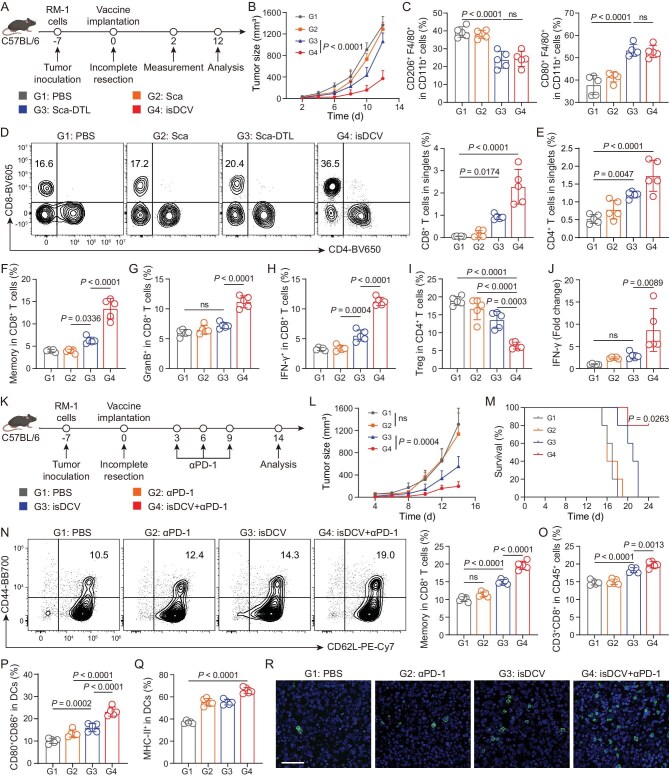
Potent antitumor immunity induced by isDCV. (A) The schematic illustration of experimental design. (B) Average tumor growth curve of mice treated with PBS, Scaffold (Sca), 3D scaffold-DTL (Sca-DTL) and BM-MNCs@3D scaffold-DTL (isDCV). (C) Quantitation of the proportion of M2-like macrophages (CD206^+^) and M1-like macrophages (CD80^+^) gating on F4/80^+^CD11b^+^CD45^+^ cells within the TME. (D) Representative flow cytometry analysis of CD8^+^ cells in CD45^+^CD3^+^ cells and quantitation of the proportion of CD8^+^ T cells in singlets in the recurrent tumor. (E–I) Quantitation of the proportion of CD4^+^ T cells in singlets (E), central memory CD8^+^ T cells in CD8^+^ T cells (F), granzyme B^+^ in CD8^+^ T cells (G), IFN-γ^+^ in CD8^+^ T cells (H) and T_reg_ CD4^+^ T cells in CD4^+^ T cells (I) in the recurrent tumor. (J) RT-qPCR showing upregulated IFN-γ in the recurrent tumor. (K) Schematic illustration of the experimental design. (L) Average tumor growth curve of mice treated with PBS, αPD-1, isDCV and combination therapy of isDCV with αPD-1 (isDCV+αPD-1). (M) Survival of mice after different treatments. (N) Representative flow cytometry analysis and quantitation of the proportion of central memory CD8^+^ T cells in CD8^+^ T cells in spleen. (O) Quantitation of the proportion of CD3^+^CD8^+^ T cells in CD45^+^ cells. (P and Q) Quantitation of the percentage of DC maturity (P), and MHC-II^+^ in DCs (Q) of dLNs. (R) Immunofluorescence images of CD8^+^ T cells in tumor. Scale bar, 50 μm. All data are expressed as mean ± SD (*n* = 5). Statistical significance was calculated via ordinary one-way ANOVA with a Tukey’s test (C, D, E, F, G, H, I, J, N, O, P, Q) or two-way ANOVA with a Tukey’s test (B, L) or log-rank (Mantel–Cox) test (M). ns, no significance.

Firstly, to evaluate the impact of the delivery strategy on the therapeutic efficacy of isDCV, we compared isDCV implantation with conventional subcutaneous injection. IsDCV implantation achieved superior tumor growth inhibition (Fig. S17), which correlated with enhanced migration of CFSE-labeled DCs to draining lymph nodes (Fig. S18), highlighting the critical role of the 3D hydrogel scaffold in effective DC-based immunotherapy. To further investigate the antitumor efficacy of the isDCV, RM-1 cells were then subcutaneously injected into the flank of mice 7 days prior to surgery. On Day 0, the primary tumor was surgically resected, leaving approximately 1% residual tumor tissue to simulate minimal residual disease within the surgical bed. Tumor tissue excised during surgery was rapidly processed into DTL. The DTL, FLT3L and GM-CSF were mixed with GelMA solution to formulate a bioink, which was mixed with BM-MNCs and bioprinted to create the isDCV. The isDCV was implanted into the post-operative tumor bed on the same day. Control groups received implantation of DTL-loaded scaffold (Sca-DTL), blank scaffold (Sca) or PBS injection into the tumor bed, followed by subsequent tumor excision for analysis on Day 12 (Fig. 5A). Tumor growth kinetics revealed no significant difference between the PBS and Sca groups (Fig. 5B). Scaffold treatment alone failed to effectively suppress tumor growth. In contrast, mice treated with isDCV exhibited significant inhibition of tumor growth (Fig. 5B). No significant body weight loss was observed in any treatment group during the therapeutic period (Fig. S19). Complete blood count and serum biochemical analyses indicated no significant differences among the groups (Figs S20 and S21). Histopathological examination [hematoxylin and eosin (H&E) staining] revealed no obvious signs of toxicity in the treated groups (Fig. S22). Finally, isDCV also significantly inhibited tumor growth in a non-surgical vaccination model (Fig. S23).

To elucidate the antitumor mechanism of isDCV, tumor-infiltrating immune cells were analyzed by flow cytometry (Fig. S24). Results demonstrated that both Sca-DTL and isDCV treatments significantly reduced the proportion of F4/80^+^CD206^+^ cells within the CD11b^+^ population, while increasing the proportion of F4/80^+^CD80^+^ cells within CD11b^+^ cells. These results indicated that the DTL component within isDCV effectively reversed the post-surgical immunosuppressive microenvironment, independent of the encapsulated cellular payload (Fig. 5C, Fig. S25). The proportion of CD8^+^ T cells within tumors was significantly higher in isDCV group compared to all other groups (Fig. 5D). Furthermore, isDCV treatment significantly increased the proportions of CD4^+^ T cells, CD8^+^ central memory T cells, granzyme B^+^ CD8^+^ T cells and IFN-γ^+^ CD8^+^ T cells compared to PBS treatment (Fig. 5E–H, Fig. S26A, B and D). Conversely, the proportion of regulatory T cells within the CD4^+^ T cell population was significantly lower in the isDCV group compared to control groups (Fig. 5I, Fig. S26C). H&E staining and terminal deoxynucleotidyl transferase dUTP nick end labeling (TUNEL) assays on tumor tissues revealed that isDCV treatment induced relevant immune responses and resulted in the highest level of tumor cell apoptosis among all groups (Fig. S27). RT-qPCR analysis of tumor tissue showed significantly elevated IFN-γ expression levels in the isDCV group compared to the PBS group (Fig. 5J). These results collectively demonstrate that isDCV promoted the infiltration of tumor-associated T cells, contributing to post-operative antitumor immunity.

### Synergy treatment with isDCV and anti-PD-1 antibodies

To further assess the application potential of isDCV, local isDCV therapy combined with intratumoral injection of anti-programmed death 1 antibody (αPD-1) was adopted to control the growth of subcutaneous recurrent tumors following resection (Fig. 5K) [47]. Tumor progression showed no significant difference between PBS and αPD-1 monotherapy groups, indicating minimal tumor suppression by αPD-1 alone (Fig. 5L). Conversely, isDCV monotherapy significantly inhibited tumor growth. Remarkably, the isDCV+αPD-1 cohort exhibited profoundly enhanced tumor suppression versus isDCV alone (Fig. 5L), concomitant with significantly prolonged overall survival versus all controls (Fig. 5M). This validates that αPD-1 reverses TME-mediated T cell suppression and potentiates DC vaccine efficacy. No substantial body weight loss occurred in treatment groups (Fig. S28).

Flow cytometric analysis of splenocytes revealed that isDCV+αPD-1 induced an ∼2-fold increase in central memory CD8⁺ T cells versus PBS controls (Fig. 5N, Fig. S29). Furthermore, this group showed increased proportions of splenic CD8⁺ T cells, CD4⁺ T cells and granzyme B⁺ CD8⁺ T cells (Fig. 5O, Fig. S30A–C), yet reduced regulatory T cells among CD4⁺ T cells (Fig. S30D). In dLNs, isDCV+αPD-1 increased mature DC proportions, and augmented DC antigen-presenting capacity versus controls (Fig. 5P and Q, Fig. S31). RT-qPCR analysis of tumor tissues demonstrated significantly upregulated TNF-α, IL-1β and CXCL-10 expression in isDCV+αPD-1 versus PBS (Fig. S32A). Serum cellular cytokine testing confirmed elevated systemic levels of TNF-α and IFN-γ (Fig. S32B). Immunohistochemical analysis revealed significantly upregulated CD8^+^ T cell infiltration within tumors following isDCV+αPD-1 treatment (Fig. 5R). The results revealed that isDCV synergizes with αPD-1 to orchestrate robust systemic immunity and potentiate post-operative antitumor memory responses.

### Inhibition of post-operative bone metastasis and tumor rechallenge by isDCV

**Figure 6.**
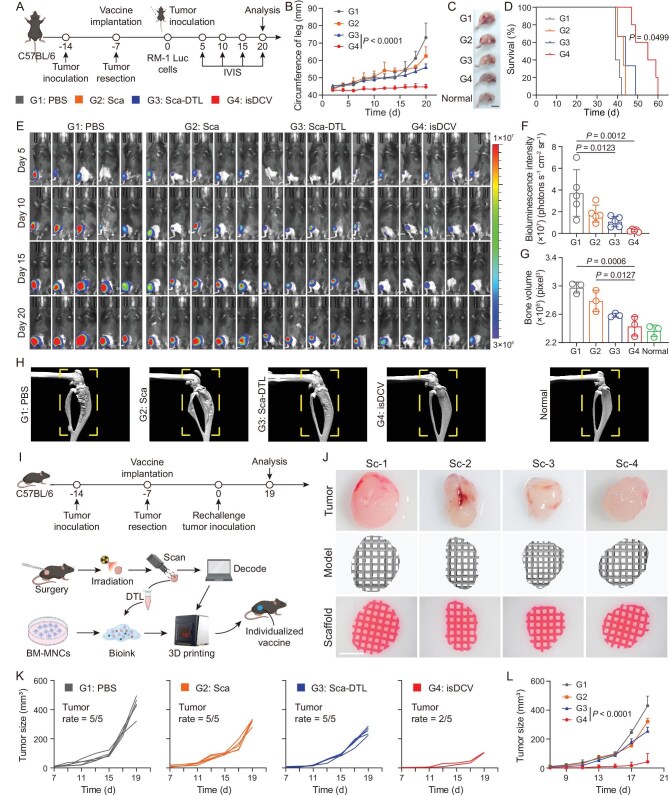
Inhibition of tumor metastasis and rechallenge after surgery by the isDCV. (A) Schematic illustration of the experimental design. (B) Mean leg circumference profile in mice treated with PBS, 3D scaffold (Sca), 3D scaffold-DTL (Sca-DTL) and BM-MNCs@3D scaffold-DTL (isDCV). (C) Representative morphology of mice legs at the site of metastasis in all groups at Day 20. Scale bar, 1 cm. (D) Survival of mice after different treatments. (E) *In vivo* bioluminescence images of RM-1 Luc tumors in all groups. (F) Bioluminescence intensity analysis of RM-1 Luc tumors on Day 20. (G) Quantitative analysis of bone volume in mice tibia and femur. (H) Representative micro-CT images of the femurs and tibias in each group. (I) Schematic illustration of post-operative tumor model and experimental design (figure made in BioRender). (J) Personalized wound filling design process. Scale bar, 400 μm. (K) Tumor growth curves of mice after different treatments. (L) Average tumor growth curve. All data are expressed as mean ± SD (B, D, F, K, L: *n* = 5; G: *n* = 3). Statistical significance was calculated via ordinary one-way ANOVA with a Tukey’s test (F, G) or two-way ANOVA with a Tukey’s test (B, L) or log-rank (Mantel–Cox) test (D).

Bone represents the most frequent site of metastasis in prostate cancer, accounting for about 90% of all metastatic cases [48]. To investigate the efficacy of isDCV in suppressing post-surgical bone metastasis, we established a post-operative bone metastasis model (Fig. 6A). Throughout the treatment period, mice in the treated groups maintained stable body weight, indicating minimal systemic toxicity (Fig. S33A). After 20 days of treatment, mice receiving isDCV exhibited significantly greater inhibition of hindlimb circumference expansion compared to other groups (Fig. 6B). Gross morphological examination revealed evident swelling and deformation in the hindlimbs of mice from PBS, Sca and Sca-DTL groups, suggestive of tumor-induced osteoblastic lesions (Fig. 6C). Additionally, treatment with isDCV significantly prolonged overall survival versus all controls (Fig. 6D). Furthermore, bioluminescent imaging demonstrated that the tumor burden, as measured by bioluminescent signal intensity from RM-1 LUC cells, was markedly suppressed in the isDCV group relative to other groups (Fig. 6E and F). Micro-computed tomography (Micro-CT) analysis of tibiae revealed extensive osteoblastic lesions in all groups except the normal group and isDCV groups (Fig. 6H). This pathological increase in bone mass likely reflects the osteoblastic factor-secreting phenotype characteristic of progressing prostate cancer bone metastases. Critically, isDCV treatment effectively mitigated this pathological increase in bone volume (Fig. 6G) and concurrently demonstrated optimal suppression of tumor progression in the bone.

To broaden the applicability of 3D printing technology for post-operative care, custom-fabricated 3D scaffolds were engineered to address surgical void-filling demands. Radical prostatectomy can induce tissue defects predisposing to post-operative complications [49]. Herein, we successfully bioprinted tumor-shaped isDCV scaffolds for surgical defect repair, recapitulating clinical scenarios of prosthetic implantation post-prostatectomy (Fig. 6I). The fabrication workflow is schematically illustrated in Fig. 6I, beginning with the resection of orthotopically implanted dorsal tumors in mice, followed by *ex vivo* tumor scanning and computer-aided design of a patient-specific 3D model; subsequent steps involve the bioprinting of isDCV into anatomically conforming scaffolds and their implantation into the resection cavity, culminating in a contralateral rechallenge with RM-1 tumor cells. Scaffolds were topographically designed based on resected tumor morphology (Fig. 6J). Notably, the isDCV treatment group exhibited superior efficacy in preventing rechallenge tumor growth versus control groups, with detectable distant tumors developing in only two mice (Fig. 6K and L). Concomitantly, cytokine testing demonstrated marked elevation of serum TNF-α and IFN-γ levels in isDCV-treated mice compared to PBS controls (Fig. S33B). Collectively, these findings demonstrate that personalized isDCV not only serves as a biocompatible void filler but also elicits potent systemic antitumor immunity to prevent rechallenge tumor progression.

## DISCUSSION

In summary, we have successfully developed a 3D hydrogel scaffold DC vaccine—isDCV—that bypasses the complex and time-consuming *ex vivo* cell culture procedures typically associated with conventional DC vaccines. This innovative *in situ* strategy used a 3D-printed GelMA scaffold co-encapsulating undifferentiated BM-MNCs, stimulating factors and personalized DTL. Upon implantation into the surgical bed, the isDCV facilitates the *in situ* differentiation of BM-MNCs into DCs, followed by their maturation stimulated by the personalized tumor antigens. This process significantly enhances their lymph node migration and subsequent robust antitumor immune responses. Studies in prostate cancer mouse models demonstrated that isDCV effectively inhibited tumor growth, suppressed metastasis and notably extended the median survival of the animals, highlighting its potential as a simple, safe and highly effective approach for cancer immunotherapy. Although the application of 3D printing bioinks is widespread [25,50], there have been few reports on the application of 3D bioprinted scaffolds for the *in situ* generation of DC vaccines.

DC vaccines exhibit substantial promise in cancer management; however, the intricate nature of *ex vivo* cell culture processes significantly constrains their widespread clinical implementation [14]. To overcome the complexities of *ex vivo* preparation and their associated clinical application limitations, researchers have been actively exploring various strategies. These strategies include: developing ‘fast DC’ technology to shorten *ex vivo* culture time [51,52]; utilizing mRNA pulsing for antigen loading [53]; and employing *in vivo* targeting strategies for endogenous DCs [54,55]. These approaches aim to simplify the preparation workflow of DC vaccines and enhance their clinical feasibility. In this context, the development of the isDCV represents a significant advancement in cancer vaccination, offering a simple and accessible platform for generating potent antitumor immunity. By bypassing the need for laborious *ex vivo* cell manipulation, this *in situ* DC vaccine strategy addresses a critical bottleneck that has historically hindered the widespread clinical translation of DC-based therapies [14,18,19].

In addition to addressing the critical bottleneck of complex *ex vivo* preparation, the isDCV further distinguishes itself through its capacity for personalization and cell viability. Its personalization, enabled by the incorporation of autologous tumor lysates and 3D printing for site-specific delivery, holds immense promise for tailoring treatment to individual patients and their specific tumor characteristics [23]. Furthermore, the ability of the hydrogel scaffold to promote long-term cell viability and provide a conducive matrix for DC differentiation and maturation within the surgical site opens new avenues for localized immunotherapy [25], potentially offering superior efficacy compared to systemic treatments, especially for solid tumors with low mutational burdens where ICB therapies often show limited success [7,8].

Looking ahead, further comprehensive investigations are warranted to fully realize the clinical potential of the isDCV. Future research should focus on optimizing the hydrogel properties, cell encapsulation densities and the precise cocktail of stimulating factors to maximize DC functionality and immune activation. Rigorous validation in larger animal models and diverse cancer types, including those less responsive to current immunotherapies, will be crucial to assess its broad applicability and long-term safety profile. Moreover, exploring more combination strategies with existing therapeutic modalities, such as immune checkpoint inhibitors or conventional chemoradiotherapy, could lead to synergistic antitumor effects and improved patient outcomes. The scalability of 3D printing for personalized vaccine fabrication and the establishment of robust manufacturing protocols will also be essential for successful clinical translation, ultimately paving the way for this promising *in situ* DC vaccine to become a cornerstone in the evolving landscape of cancer immunotherapy.

## Ethics

All animal experiments strictly followed the guidelines approved by the Institutional Review Board of Shenzhen Bay Laboratory (approval number: AERL202501).

## Supplementary Material

nwag037_Supplemental_File
