# Supplementary material for: In situ generation of dendritic cell vaccines in 3D printing scaffolds for cancer post-surgical therapy
Source: Natl Sci Rev. 2026 Jan 20;13(5):nwag037. doi: 10.1093/nsr/nwag037 (PMC12912718; doi:10.1093/nsr/nwag037)
Supplement: nwag037_Supplemental_File [file nwag037_supplemental_file.pdf]

Supporting Information

In Situ Generation of Dendritic Cell Vaccines in 3D Printing Scaffolds for Cancer Post-Surgical Therapy

Lefan Chen[1,2,#], Yangtao Xu[2,3,#], Xiao Hu[2,#], Yuanwei Pan[2,4], Peng She[5], Xiaoyuan Chen[4,6,7], Qiyu Peng[8], Qi Li[1,*], and Lang Rao[2,*]

[1] Department of Urology, The First Affiliated Hospital of Zhengzhou University, Zhengzhou 450052, China.

[2] Institute of Chemical Biology, Shenzhen Bay Laboratory, Shenzhen 518132, China.

[3] Cancer Center, Renmin Hospital of Wuhan University, Wuhan 430060, China.

[4] Department of Diagnostic Radiology, Yong Loo Lin School of Medicine, National University of Singapore, Singapore 119074, Singapore.

[5] Department of Orthopedics, The Seventh Affiliated Hospital of Sun Yat-Sen University, Shenzhen 518000, China.

[6] Nanomedicine Translational Research Program, Yong Loo Lin School of Medicine, National University of Singapore, Singapore 117597, Singapore.

[7] Institute of Molecular and Cell Biology, Agency for Science, Technology, and Research (A*STAR), Singapore 138673, Singapore.

[8] Institute of Biomedical Engineering, Shenzhen Bay Laboratory, Shenzhen 518132, China.

[#] L.C., Y.X., and X.H. contributed equally to this work.

* Corresponding e-mail: richee@zzu.edu.cn (Q.L.); and lrao@szbl.ac.cn (L.R.).

Materials and Methods

Materials and reagents

Lipophilic stain 1,1'-dioctadecyl-3,3,3',3'-tetramethylindodicarbocyanine perchlorate (DiD) and the intracellular dye carboxyfluorescein succinimidyl ester (CFSE) were purchased from Thermo Fisher Scientific Inc. All other chemicals were purchased from Sigma-Aldrich, unless otherwise noted.

Mice

C57BL/6 male mice aged 6-10 weeks were obtained from Gempharmatech Co., Ltd. The animal study received approval from the Institutional Review Board of Shenzhen Bay Laboratory (AERL202501) and was conducted in alignment with the principles of guidelines for animal experiment protection.

Cells

RM-1 cells were cultured in Dulbecco's Modified Eagle Medium (DMEM) obtained from Invitrogen. To create RM-1 cells tagged with luciferase, the cells underwent transfection using vectors that included both luciferase and a puromycin resistance gene. The RM-1 Luc cells were maintained in Roswell Park Memorial Institute 1640 (RPMI 1640, Invitrogen) culture medium. Cells were incubated in an atmosphere containing 5% CO2 and were supplemented with 10% fetal bovine serum (FBS), along with 100 U/mL of penicillin and 100 μg/mL of streptomycin, all of which were sourced from Invitrogen.

Bone marrow-derived dendritic cells (BMDCs) were prepared using the following procedure: 1×10^6 bone marrow cells were placed in a 10 cm culture dish and mixed with 10 mL of complete medium, which consisted of RPMI 1640 supplemented with glutamine, penicillin, and streptomycin, 10% heat-inactivated fetal bovine serum (all from Invitrogen), as well as Flt-3 ligand (FLT3L) at 100 ng/mL (Abcam, ab270071) and granulocyte-macrophage colony-stimulating factor (GM-CSF) at 20 ng/mL (Peprotech). On the third day, medium was added, and on days 6 and 8, the medium was partially replaced. Finally, on day 10, non-adherent cells from the supernatant along with the loosely-adherent cells that had been gently washed with PBS were collected for subsequent experiments.[1]

Bone marrow-derived macrophages (BMDMs) were obtained through the following process. C57BL/6 mice were euthanized to isolate bone marrow cells from their leg bones. These cells were then cultured in RPMI 1640 medium enriched with 10% heat-inactivated FBS and 1%

antibiotics and were allowed to differentiate for 7 days in the presence of macrophage colony-stimulating factor (M-CSF) at 20 ng/mL (Peprotech).[2]

Preparation and characterization of GelMA hydrogel

Methacrylic anhydride-modified gelatin (GelMA) was produced based on methods described in earlier studies.[3] In this procedure, 10.0 g of gelatin (Gel) was dissolved in 100 mL of PBS at a temperature of 55 °C while stirring continuously, resulting in a gelatin solution. In a separate container, 8.5 mL of methacrylic anhydride (MA) was combined with 20 mL of PBS to create MA solution. This MA solution was then added dropwise to the gelatin solution while stirring, allowing the reaction to proceed for three hours in the dark at 55 °C. The pH of the mixture was kept around 8.5 during the entire process. To stop the reaction, 600 mL of distilled deionized water (ddH_2O) was added. Following this, the resulting solution was dialyzed against ddH_2O for three days. After dialysis, the solution was freeze-dried to yield GelMA.

The GelMA was dissolved in PBS (10%, w/v) containing 0.5% (w/v) photo-initiator lithium phenyl (2,4,6-trimethylbenzoyl) phosphinate (LAP). After 1 min of blue light (405 nm) exposure, the GelMA solution transformed to crosslinked GelMA hydrogels. The GelMA was examined by ^1H NMR (Bruker 400 MHz Advance, Switzerland) in D_2O to confirm the methylacryloyl modification.

The photo-curing rheological characteristics of GelMA were evaluated using a TA-DHR-2 rheometer (TA Instruments, USA) equipped with parallel-plate geometry (P20 TiL, 20-mm diameter) and an OmniCure Series 2000 (405 nm, 30 mW cm^{-2}) at a temperature of 37 °C. The storage modulus (G') and loss modulus (G'') were recorded over time, with the gel point identified as the moment when the storage modulus exceeded the loss modulus. Additionally, the microstructure of the lyophilized GelMA hydrogel was analyzed using scanning electron microscopy (SEM) (FEI Quanta 200, FEI Company, Czech Republic).

Porosity measurement

First, isDCV were soaked in deionized water for 1-2 hours. Afterward, they were positioned on Kimwipe paper to dry one side of the gel, allowing the water within the pores to escape *via* capillary action. The weights of the gels were recorded both before (Wi) and after (Wf) the wicking assay using an electronic scale. The swelling rate was then calculated using the following formula:

$$\text{Porosity}(\%) = \frac{Wi - Wf}{Wi} \times 100 \qquad (1)$$

Swelling performance characterization: The hydrogel samples were freeze-dried first, and then soaked in PBS at 37°C for 24 h. The wet weight (Ws) of the hydrogel was measured by electronic balance. The swelling rate is calculated as follows:

$$\text{Swelling rate}(\%) = \frac{Ws - Wd}{Wd} \times 100 \tag{2}$$

Where *Ws* is the moisture content of the hydrogel and *Wd* is the dry weight of the hydrogel after freeze-drying.

Characterization of mechanical properties: compressive stress-strain of hydrogel was measured by AGS-V universal tester. A compression test sample with a height of 3 mm and a diameter of 10 mm was prepared by photocuring. Compression is performed at a rate of 0.5-1 mm/min until the stress change exceeds 1% or the sample breaks.

3D bioprinting of isDCV bioink

Prior to printing, 5 mg of the LAP is added to 1 mL of GelMA solution, followed by thorough shaking and subsequent incubation at 37°C for 1h. Subsequently, 1 × 10^7 bone marrow mononuclear cells (BM-MNCs) and 200μg of tumor lysates enriched with Damage-Associated Molecular Patterns (DTL) are introduced into 1 mL of the GelMA solution with LAP, and gentle mixing is achieved by pipetting, resulting in the production of isDCV bioink.

A digital light processing printer (nanoArch S240, BMF Material Technology Inc, China), which uses a 405 nm laser, was utilized for the 3D bioprinting process. The 3D CAD models of the intended designs were developed using SolidWorks software. The parameters for 3D bioprinting can be found in Supporting Table S1. Following the printing process, the resulting samples were analyzed with digital microscopy (Dino-Lite, AnMo Electronics Corporation). After a 7-day incubation period, the samples underwent staining with calcein-AM and PI for 20 minutes before being evaluated using confocal laser scanning microscopy (CLSM).

In addition, the cell morphology in the scaffolds was observed by SEM. The specific steps are as follows:

Step 1: Mix BM-MNCs with hydrogel at a cell density of 1 × 10^7/mL.

Step 2: Immediately perform 3D printing after adding LAP, and culture the BM-MNCs@scaffolds in inactivated serum medium.

Step 3: After 3 days, remove the scaffolds and wash them three times with 1 mL PBS medium.

Step 4: Discard PBS and fix the samples with 2.5-3.0% glutaraldehyde for 2.5-3h.

Step 5: Discard glutaraldehyde and perform ethanol dehydration with gradients of 30%, 50%, 75%, 80%, 95%, and 100% (twice) for 15 minutes each.

Step 6: Replace with isopentyl acetate in a glass bottle for more than 30 minutes.
Step 7: After drying, the hydrogels were manually fractured with clean, precooled forceps to obtain smooth cross-sections, which were then examined under SEM.
Step 8: Utilize the software Photoshop 2022 (Adobe Systems Incorporated, USA) to color the DCs.

Confocal Z-stack imaging of DCs inside hydrogels

Dendritic cells were first stained with DAPI to label cell nuclei. Briefly, DCs were incubated with DAPI, washed thoroughly with PBS to remove excess dye, and then directly mixed with the hydrogel precursor solution. The DAPI-labeled cells were subsequently encapsulated through 3D printing to form cell-laden hydrogel constructs. After printing, the constructs were incubated for 2 h at 37 °C to allow stabilization of the cellular microenvironment.

Confocal imaging was performed using a Zeiss LSM 900 laser scanning confocal microscope. Z-stack images were acquired to examine the vertical distribution of cells within the hydrogel. Image acquisition and reconstruction were conducted using Zeiss ZEN software to visualize the presence of DCs within the internal structure of the printed hydrogels.

Analysis of cell viability within the scaffold

We selected four regions within the scaffold images and adjusted the grayscale uniformly for each region using ImageJ.[4] We then separately measured the fluorescence intensity of green and red colors, and calculated the ratio of green fluorescence intensity to the total fluorescence intensity to represent cell viability.

In vivo degradation of isDCV

To quantitatively characterize the *in vivo* degradation kinetics of the isDCV, constructs were implanted subcutaneously in C57BL/6 mice. Scaffolds were explanted at 72-hour intervals, lyophilized, and subjected to gravimetric analysis (dry mass) alongside macroscopic imaging to assess degradation progression.

Isolation of tumor lysates

Tumor lysates were prepared from cultured cancer cells grown in 10 cm dishes. For the preparation of irradiation-induced tumor lysate (DTL), cells were exposed to a single dose of 20 Gy irradiation using a Rad Source RS2000pro system. For the preparation of tumor lysate without irradiation (TL), the same procedure was followed except that the irradiation step was omitted. Immediately after irradiation or under identical conditions for TL preparation, the original culture medium was replaced with serum-free DMEM. After incubation for 2 h, both the cells and the culture medium were collected and subjected to at least five repeated freeze-thaw

cycles, followed by sonication to ensure complete cell lysis. The lysates were then centrifuged at 12,000 rpm for 5 min, and the resulting supernatant containing tumor antigens was collected and used as TL or DTL, respectively.[5] In addition, ATP content was measured by ATP detection kit (Beyotime Biotech, China), the contents of CRT and HMGB-1 were detected by ELISA detection kits (Dakovia Biotech).

For the lysis of tumor tissues, samples were obtained from mice that had the relevant tumors. The tissues were exposed to a single dose of 20 Gray radiation using a Rad Source RS2000pro, then minced into small fragments and homogenized. Following this, the mixture was rinsed through cell strainers with ultrapure water. The resulting tissue cells underwent a minimum of five freeze-thaw cycles accompanied by sonication. Afterward, the samples were centrifuged at 12,000 rpm for 5 minutes, and the supernatant was collected as DTL.

Ex vivo maturation of BMDCs

On the 10th day of BMDCs culture, BMDCs were collected and reseeded into a 12-well plate at 1 × 10^6 cells/mL. Each group was treated with PBS, 0.1 μg/mL LPS, 200 μg/mL TL, or 200 μg/mL DTL. After 18 hours, the BMDCs were collected for flow cytometric analysis. In addition, the supernatant was collected for ELISA analysis (Invitrogen).

Cytotoxicity of DTL

The cytotoxicity test was performed using the CCK8 assay. First, BMDCs were seeded onto 96-well polystyrene plates at 10,000 cells in 100 μL of medium each well. After a 12 hours incubation, the cells were treated with 200 μg/mL DTL in the medium for durations of 1 day, 4 days, and 7 days. Following this treatment, 100 μL of CCK8 solution was added, and the cells were incubated for an additional 2 hours. The evaluation of cell viability was conducted by assessing the absorbance at 450 nm with the use of a microplate reader.

Survival and maturation of BMDCs in 3D scaffold

On day 10 of BMDCs culture, 1 × 10^6 BMDCs were collected, resuspended, and mixed with either: 1) a GelMA solution containing DTL (200 μg/mL), FLT3L (100 ng/mL), and GM-CSF (20 ng/mL), then bioprinted into scaffold constructs (DCs@Sca-DTL); 2) a GelMA solution containing FLT3L (100 ng/mL) and GM-CSF (20 ng/mL), then bioprinted into scaffold constructs (DCs@Sca); or 3) a GelMA solution containing FLT3L (100 ng/mL) and GM-CSF (20 ng/mL), which was bioprinted into bulk hydrogel constructs (DCs@HG). All groups were subsequently cultured *in vitro* for an additional 7 days. Following this culture period, cells were retrieved for flow cytometry analysis

by enzymatically digesting the hydrogel constructs using a commercial hydrogel lysis solution (EFL-GM-LS-001, 0.3 mg/mL).

In vivo differentiation of BM-MNCs

To investigate the differentiation of BMDCs, on day 0 of BM-MNCs isolation, 1 × 10^6 BM-MNCs were collected, resuspended, and mixed with either: 1) a GelMA solution containing DTL (200 μg/mL), FLT3L (100 ng/mL), and GM-CSF (100 ng/mL), bioprinted into scaffold constructs (isDCV); or 2) untreated GelMA solution, bioprinted into scaffold constructs (BM-MNCs@Sca). Subsequently, four healthy C57BL/6 (male, 6 weeks) were allocated to each experimental group. The fabricated scaffolds were surgically implanted into postoperative tumor cavities within these mice. Scaffolds were explanted at postoperative days 1, 4, 7, and 10 for flow cytometric analysis. The flow cytometric analysis procedure is as follows:

Scaffolds were removed from the mice and lysed by GelMA lysate (EFL-GM-LS-001, 0.3 mg/mL). Isolated cells were first determined Live or Dead using Zombie AquaTM Dye (BioLegend) for 10 min at 4 °C. Then, cells were blocked at 4°C for 5 minutes and then stained with following antibodies: CD11c-PE (clone HL3; BD Biosciences), CD80-BV421 (clone 16-10A1; BD Biosciences), CD86-BV650 (clone GL1; BD Biosciences), MHC-II I-A/I-E-PerCP-Cy7 (clone M5/114.15.2; BioLegend). Finally, the samples were analyzed using a CytoFLEX flow cytometer (Beckman Coulter).

RNA sequencing and bioinformatic analysis

Absolute quantitative transcriptome sequencing was performed by the Institute of Hydrobiology, Chinese Academy of Sciences. Briefly, BM-MNCs were loaded into isDCV. Subsequently, 3 healthy C57BL/6 (male, 6 weeks) mice were allocated to each group, then, the scaffolds were removed on day 10. Total RNA was extracted, and mRNA was enriched using oligo(dT) magnetic beads. The purified mRNA was subsequently fragmented into ~300 bp fragments using lysis buffer. First-strand cDNA synthesis was carried out using the fragmented mRNA as a template, followed by purification, end repair, and PCR amplification to generate sequencing libraries, ensuring a final library concentration greater than 2 nM. Sequencing was conducted on the Illumina platform.

The sequencing data were analyzed using R software (version 4.2.0). Differential expression analysis of genes was performed with the DESeq2 package, employing a threshold of FDR < 0.05. Heatmaps were created utilizing the Pheatmap package. For Gene Ontology (GO) functional

enrichment analysis, the ClusterProfiler package was used, while Gene Set Enrichment Analysis (GSEA) was conducted using GSEA software from the Broad Institute.

Flow cytometric analysis of DC migration to lymph nodes

BMDCs were labeled with DiD dye at 37°C for 5 minutes, followed by a 10-minute incubation at 4°C. Subsequently, the DCs were washed with PBS and seeded onto scaffolds. In separate experiments using an independent cohort of mice, DC migration was further validated by intracellular CFSE labeling. Briefly, BMDCs were incubated with CFSE (5 μM) at 37 °C for 10 min, quenched with complete medium, washed with PBS, and seeded onto scaffolds. 4 healthy C57BL/6 (male, 6 weeks) mice were allocated to each treatment group (DCs@3D Sca, DCs@3D Sca-TL, and DCs@3D Sca-DTL). The flow cytometric analysis procedure is as follows:

Lymph nodes were removed from the mice and ground through a 70-um filter to obtain lymphocytes. Isolated cells were first determined Live or Dead using Zombie AquaTM Dye (BioLegend) for 10 min at 4°C. Then, cells were blocked at 4°C for 5 minutes and then stained with following antibodies: CD11c-PE (clone HL3; BD Biosciences), CD80-BV421 (clone 16-10A1; BD Biosciences), CD86-BV650 (clone GL1; BD Biosciences), MHC-II I-A/I-E-PerCP-Cy7 (clone M5/114.15.2; BioLegend). Finally, the samples were analyzed using a CytoFLEX flow cytometer.

***In vivo* systemic toxicity and safety evaluation**

To assess the *in vivo* toxicity of isDCV, we conducted subcutaneous injections of PBS or the scaffold in C57BL/6 mice. On day 30 after transplantation, the mice were euthanized, and blood samples were taken for comprehensive blood analysis and biochemical evaluation using a blood analyzer (7080, HITACHI, Japan). The primary organs from each group, including the heart, liver, kidneys, lungs, and spleen, were harvested for H&E staining to examine any systemic pathological alterations.[2]

Immunohistochemistry

Tumor sections underwent a deparaffinization and rehydration process, followed by antigen retrieval with sodium citrate. Endogenous peroxidase activity was subsequently inhibited. The sections were then treated with the working buffer from the TUNEL kit, a CD8 antibody at a dilution of 1:1000, and secondary antibodies. Imaging was performed with a Pannoramic MIDI scanner (3DHISTECH).

Quantitative real-time PCR

Total RNA was isolated from the cells with Trizol Reagent. cDNA synthesis was performed using the PrimeScript RT Reagent Kit from Takara. For the quantification of mRNA expression, RT-

qPCR was conducted on the cDNAs with the SYBR Premix Ex Taq RT-PCR kit (Takara). ACTIN was utilized as a housekeeping control in the mouse samples. Data collection was carried out using the Roche LC 480 system. The fold change in expression was determined following the $2^{-\Delta\Delta Ct}$ method. Primer sequences for all genes can be found in Supporting Table S2.

***In vivo* tumor models and treatments**

In the RM-1 tumor recurrence model experiment, 5×10^5 RM-1 cells were implanted into the right side of the C57BL/6 mouse flank (male, 6 weeks). On day 7 post-inoculation, surgical resection of tumor tissues was performed, retaining approximately 1% residual tumor tissue to mimic microscopic residual disease within the surgical bed. Subsequently, scaffolds fabricated using 3D printing technology were implanted at the tumor resection site. The experimental mice were divided into four groups: group 1 received a subcutaneous injection of PBS at the surgical site (PBS); group 2 received a scaffold loaded with FLT3L (100 ng/mL), and GM-CSF (100 ng/mL) (Sca); group 3 received a scaffold co-loaded with DTL (200 μg/mL), FLT3L (100 ng/mL), and GM-CSF (100 ng/mL) (Sca-DTL); group 4 received a scaffold co-loaded with BM-MNCs (1×10^6 cells per mouse), DTL (200 μg/mL), FLT3L (100 ng/mL), and GM-CSF (100 ng/mL) (isDCV).

In the study examining the synergistic effect of PD-1 blockade on the recurrence of RM-1 tumors post-surgery, a total of 5×10^5 RM-1 cancer cells were implanted in the right flank of male C57BL/6 mice, aged 6 weeks. Seven days following the injection, a surgical procedure was performed to excise the tumor tissues, leaving approximately 1% of the residual tumors to replicate the presence of microtumors in the surgical site. Subsequently, scaffolds fabricated using 3D printing technology were implanted at the tumor resection site. The first group received subcutaneous injection of PBS at the surgical site (PBS), the second group received intratumoral injections of αPD-1 antibody into the residual tumor on postoperative days 3, 6, and 9 (70 μg per mouse) for a total of three doses (αPD-1), the third group received a scaffold co-loaded with BM-MNCs (1×10^6 cells per mouse), DTL (200 μg/mL), FLT3L (100 ng/mL), and GM-CSF (100 ng/mL) (isDCV), the forth group received isDCV implantation at the resection site combined with intratumoral injections of αPD-1 into the residual tumor on postoperative days 3, 6, and 9 (70 μg per mouse) for a total of three doses (isDCV+αPD-1).

For the bone metastasis of RM-1 tumor model, the C57BL/6 (male, 6 weeks) were divided into 4 groups, each consisting of 5 mice. 5×10^5 RM-1 tumor cells were first implanted subcutaneously on the right flank of mice on day -14. On day -7, the tumor was excised. Subsequently, scaffolds fabricated using 3D printing technology were implanted at the tumor resection site. Then, group 1 received a subcutaneous injection of PBS at the surgical site (PBS);

group 2 received a scaffold loaded with FLT3L (100 ng/mL), and GM-CSF (100 ng/mL) (Sca); group 3 received a scaffold co-loaded with DTL (200 μg/mL), FLT3L (100 ng/mL), and GM-CSF (100 ng/mL) (Sca-DTL); group 4 received a scaffold co-loaded with BM-MNCs (1×10^6 cells per mouse), DTL (200 μg/mL), FLT3L (100 ng/mL), and GM-CSF (100 ng/mL) (isDCV). On day 0, RM-1 Luc cells (3×10^5 in 10 μL) were injected into the proximal tibias of C57BL/6 mice to create a model of bone metastasis. Tumor progression in the femurs was monitored at regular intervals using IVIS (Caliper, Perkinelmer). Additionally, the leg circumference (calculated as circumference $= 2 \times \pi \times \text{width} + 4 \times (\text{length} - \text{width})$).

For the personalized postoperative rechallenge prevention model, healthy C57BL/6 (male, 6 weeks) mice were selected. The mice were divided into 4 groups, each consisting of 5 mice. 5×10^5 RM-1 tumor cells were first injected subcutaneously on the right flank of mice on the day -14. On the day -7 the tumor was excised. Based on the size and shape of the tumor, a scaffold model was designed, and then the personalized scaffolds were 3D bioprinted and transplanted at the excision site of the tumor. Then group 1 received a subcutaneous injection of PBS at the surgical site (PBS); group 2 received a scaffold loaded with FLT3L (100 ng/mL) and GM-CSF (100 ng/mL) (Sca); group 3 received a scaffold co-loaded with DTL (200 μg/mL), FLT3L (100 ng/mL), and GM-CSF (100 ng/mL) (Sca-DTL); group 4 received a scaffold co-loaded with BM-MNCs (1×10^6 cells per mouse), DTL (200 μg/mL), FLT3L (100 ng/mL), and GM-CSF (100 ng/mL) (isDCV). On day 0, to study the prevention effect on tumor rechallenge, 5×10^5 RM-1 cells were subcutaneously injected into the left dorsal region of above mice. The mice's body weight and tumor volume were monitored every two days.

Micro-CT

Femurs and tibias were observed under micro-CT (AMIL Preclinical CT Imaging System). The micro-CT scans were performed with the following parameters: Spatial resolution: 30 μm; Voxel size: 20 μm; X-ray tube voltage: 60 kV; X-ray tube current: 600 μA; Integration time per projection: 100 ms; Total scan duration (Acquisition time): 180 s; Angular step: 5 projections per degree; Field of View: 60 mm. Images and bone volume fraction were acquired by Image J.[4]and analysed using Bone J.[6]

Flow cytometry analyses of mice after treatments

The tumor tissue was minced and suspended in a digestion solution (Serum-free 1640 medium with 25 U/mL DNase I, 0.2 mg/mL Collagenase and 0.1 mg/mL hyaluronidase; Sigma) in gentleMACS(TM) C Tube, with 5 mL digestion solution per tube. The m-imp tumor-01-01 was

run on an automated processor (GentleMACS Dissociator Instruments) for 37 seconds each time, and then shaken at 37°C at 150 rpm for 40 minutes. After digestion, the cell suspension was filtered through a 70 μm mesh into a 50 mL centrifuge tube, and the supernatant was removed by centrifugation, and 5mL of red blood cell lysis solution (Solarbio) was added to each tube to remove red blood cells. As for tissues of lymph nodes, they were ground through a 70-um filter to obtain lymphocytes and splenocytes. Then red blood cell lysis solution was added to splenocytes to remove red blood cells. Isolated cells were initially assessed for viability using Zombie AquaTM Dye (BioLegend) at 4 °C for 10 minutes. After that, the cells were blocked to prevent non-specific binding. Subsequently, they were stained with the following antibodies: CD45-APC-Cy7 (clone 30-F11; BD Biosciences), CD3-FITC (clone 145-2C11; BD Biosciences), CD11c-BV605 (clone N418; BioLegend), CD4-BV650 (clone RM4-5; BioLegend), CD8-BV605 (clone 53-6.7; BD Biosciences), CD80-BV421 (clone 16-10A1; BD Biosciences), CD86-BV650 (clone GL1; Invitrogen/BD Biosciences), CD44-BB700 (clone IM7; BD Biosciences), and CD62L-PE-Cy7 (clone MEL-14; BD Biosciences).

Prior to the staining of intracellular proteins, tumor cells that had been labeled with surface markers were fixed and permeabilized using the Fixation/Permeabilization Kit (BD, USA). Following this, the cells were incubated with intracellular antibodies: Foxp3-PE (clone MF-14; BioLegend), IFN-γ-BV421 (clone XMG1.2; BD Biosciences), and GranzymeB-APC (clone QA16A02; BioLegend) for a duration of 30 minutes. For the multicolor analysis, a control with single-stained beads for each fluorochrome was prepared to facilitate compensation in every experiment. Lastly, the samples were analyzed using a CytoFLEX flow cytometer.

Cytokine detection

14th day after vaccine transplantation, the tumor tissue was removed for downstream analysis, and C57BL/6 mouse serum was collected to measure the levels of IFN-γ, and TNF-α using corresponding ELISA kits (all from Invitrogen) according to the manufacturer's instructions.

Statistical analysis

All results are presented as mean ± standard deviation (S.D.). The unpaired two-tailed t-test was used for two group comparisons and ordinary one-way (or 2way) ANOVA with a Tukey's test were used for multiple group comparisons. The log-rank (Mantel–Cox) test was used to determine the mouse survival benefit. All statistical analyses were performed with the assistance of Prism 9.0 software (GraphPad). The Figure 1A, 1B, 2A, 2J, 2L, 3A, 4A, and 6I were created with BioRender.com.

References

1. Lutz MB, Kukutsch N, Ogilvie ALJ *et al.* An advanced culture method for generating large quantities of highly pure dendritic cells from mouse bone marrow. *J Immunol Methods* 1999; **223**: 77-92.
2. Rao L, Wu L, Liu Z *et al.* Hybrid cellular membrane nanovesicles amplify macrophage immune responses against cancer recurrence and metastasis. *Nat Commun* 2020; **11**: 4909.
3. Xu Y, Zhu W, Wu J *et al.* 3D-printed dendritic cell vaccines for post-surgery cancer immunotherapy. *Adv Funct Mater* 2024; **34**: 2400507.
4. Schindelin J, Arganda-Carreras I, Frise E *et al.* Fiji: an open-source platform for biological-image analysis. *Nat Methods* 2012; **9**: 676-82.
5. Zhao Z, Ledezma DK, Affonso de Oliveira JF *et al.* A cowpea mosaic virus adjuvant conjugated to liposomes loaded with tumor cell lysates as an ovarian cancer vaccine. *Nat Commun* 2025; **16**: 5047.
6. Domander R, Felder A, Doube M. BoneJ2 - refactoring established research software [version 2; peer review: 3 approved]. *Wellcome Open Res* 2021; **6**: 37.

Supplementary Figures and Tables

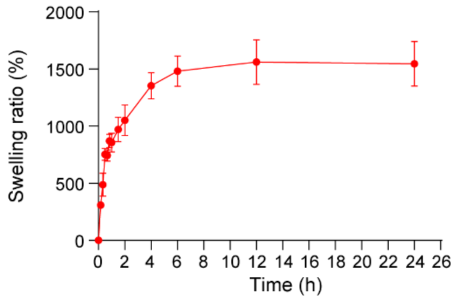

Figure S1. The swelling ratio of the GelMA hydrogel was measured at the indicated time points after immersion in PBS. All data are expressed as mean ± S.D. (n = 3).

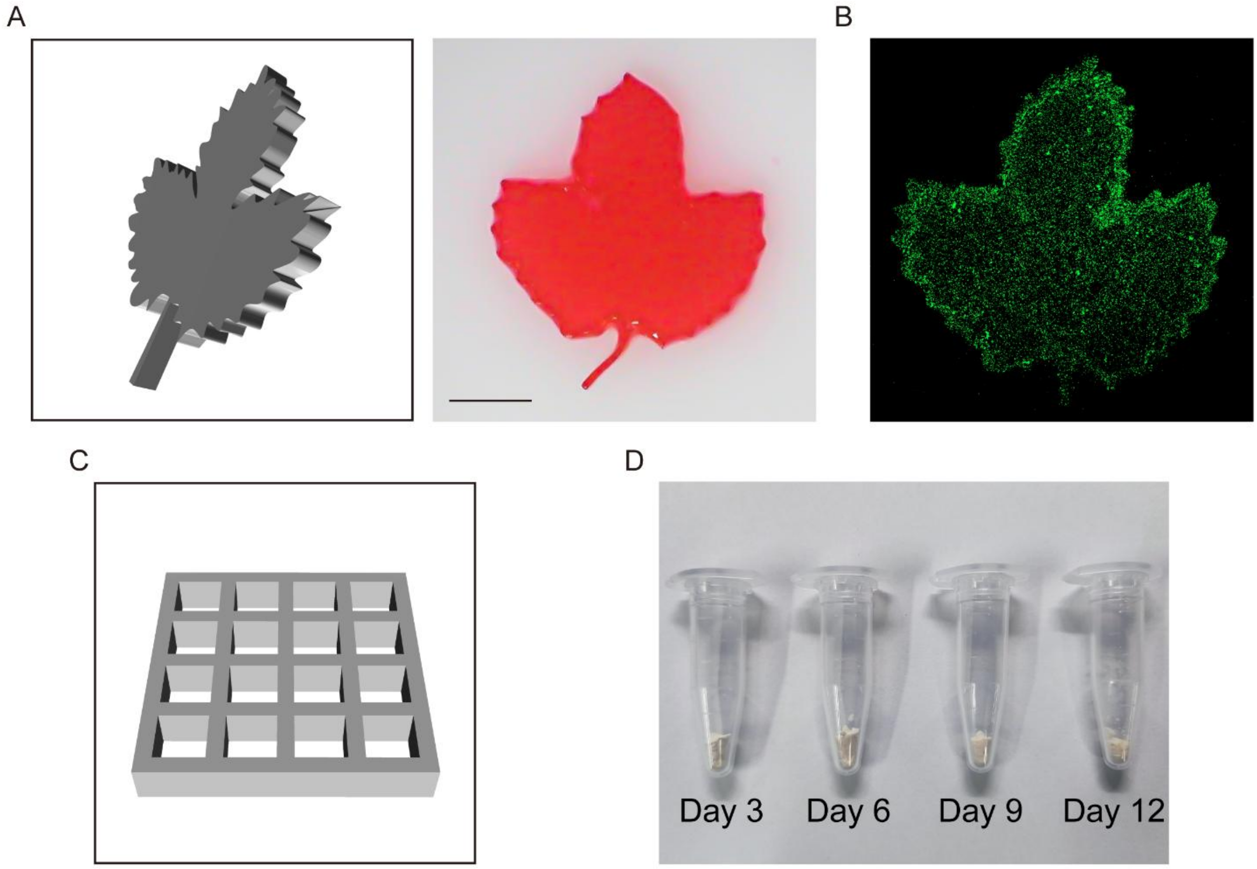

Figure S2. Synthesis and Characterizations of 3D bioprinted DC scaffold. A) DCs@scaffold printed by maple leaf model. Scale bar, 500 μm. B) 3D bioprinting DCs scaffold of maple leaf model. C) Model of scaffold. D) Characteristic of scaffold after extraction and freeze drying.

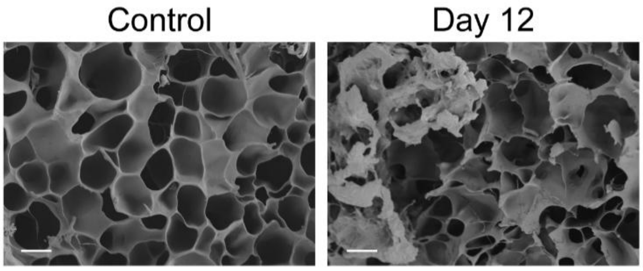

Figure S3. Representative SEM images of the GelMA hydrogel before implantation (Control) and after 12 days of *in vivo* degradation (Day 12). Scale bars, 10 μm.

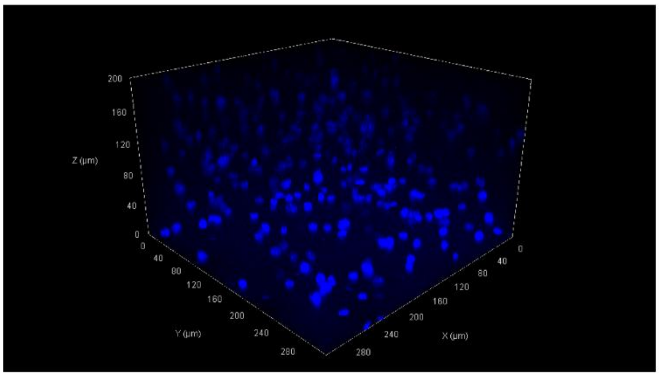

Figure S4. Representative confocal z-stack images of DAPI-labeled DCs (blue) encapsulated in the hydrogel.

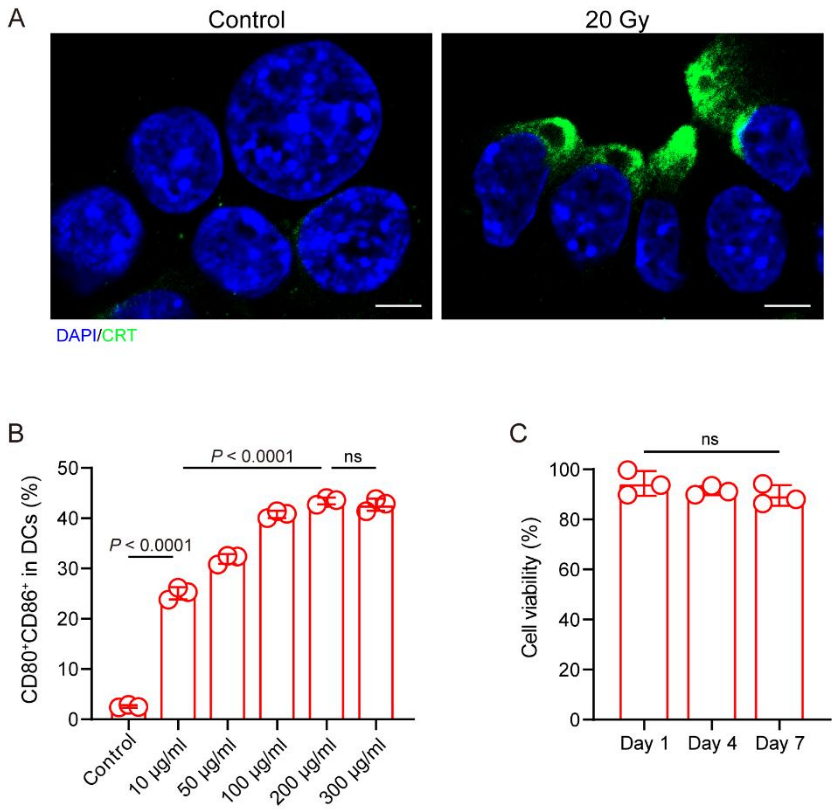

Figure S5. Characterization of DTL and its immunomodulatory ability to BMDC. A) Immunofluorescence images of RM-1 cells after different treatments. Scale bars, 5 μm. B) Immunomodulation effect of different concentrations of DTL on BMDCs. C) Effect of 200 μg/ml DTL on the viability of DCs. All data are expressed as mean ± S.D. (n = 3). Statistical significance was calculated *via* ordinary one-way ANOVA with a Tukey's test.

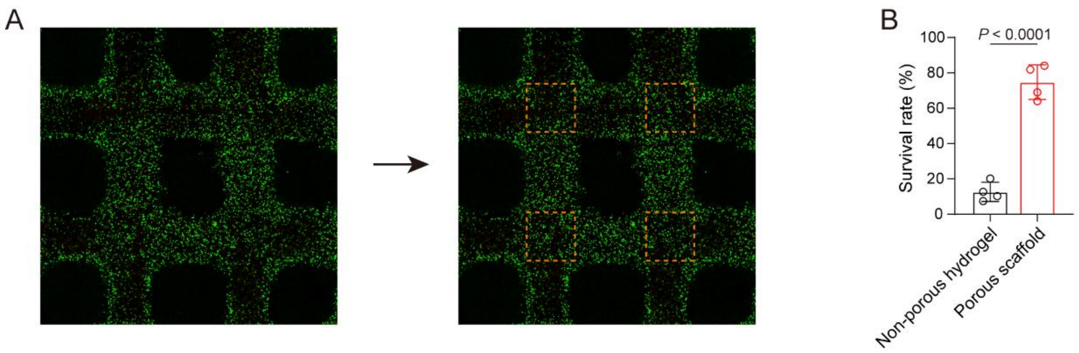

Figure S6. The survival rate of DCs on day 7. A) Take four parts from the scaffold. B) The survival rate of DCs in non-porous hydrogel and porous scaffold. All data are expressed as mean ± S.D. (*n* = 4). Statistical significance was calculated *via* unpaired two-tailed t-test.

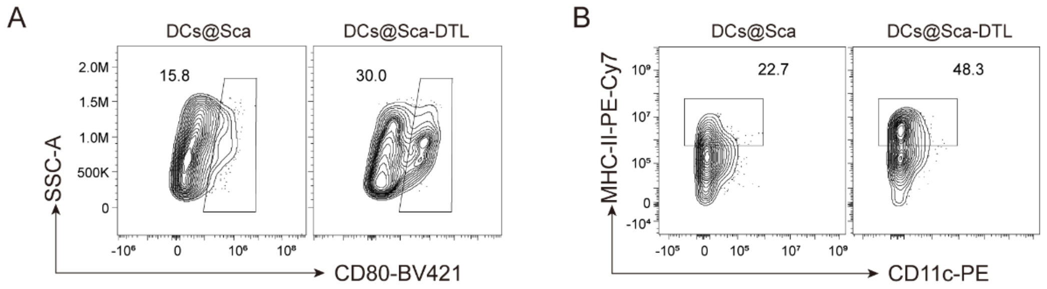

Figure S7. Flow cytometry analysis of DCs in hydrogel. A,B) Representative flow cytometry analysis of CD80+ (A), and MHC-II+ (B) in DCs.

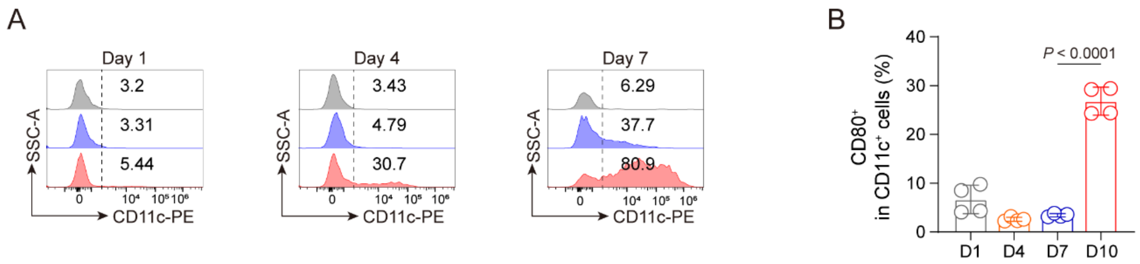

Figure S8. Flow cytometry analysis of cells in GelMA hydrogel scaffolds co-loaded with BM-MNCs, DTL and GM-CSF (isDCV). A) Representative flow cytometry analysis of DCs in live cells in 3D scaffolds co-loaded with BM-MNCs (BM-MNCs@Sca), and isDCV on day 1, 4, and 7. B) Quantitation of the proportion of $CD80^+$ DCs in the DCs in isDCV on day 1, 4, 7 and 10. All data are expressed as mean ± S.D. (n = 4). Statistical significance was calculated *via* ordinary one-way ANOVA with a Tukey's test.

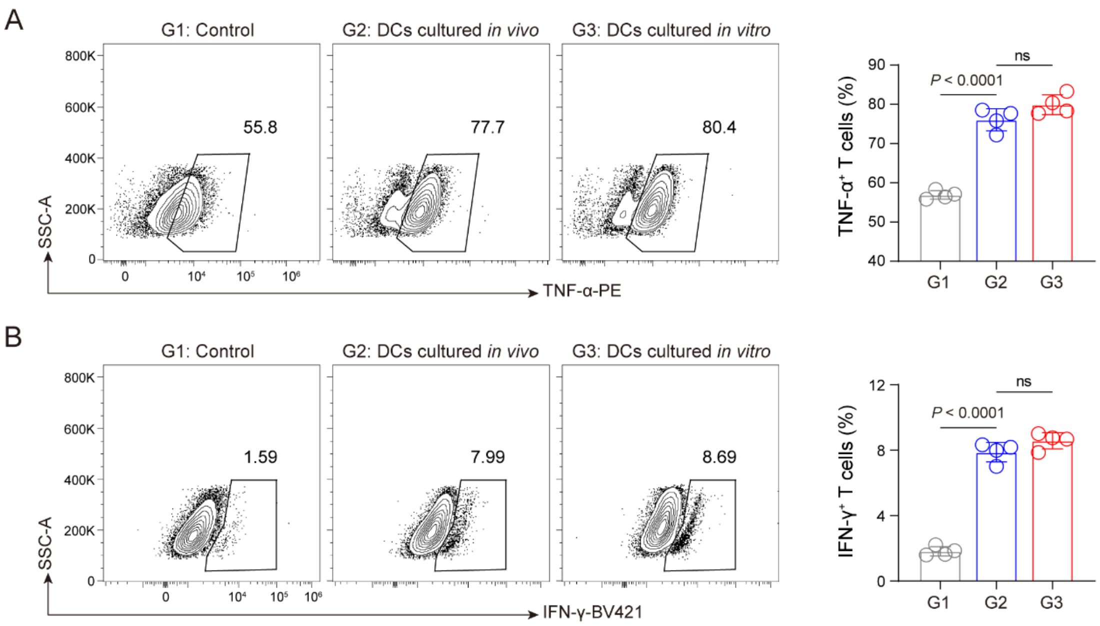

Figure S9. Representative flow cytometry analysis and quantitation of the proportion of TNF-α^+ and IFN-γ^+ T cells after co-culture with DCs from the indicated groups. All data are expressed as mean ± S.D. (n = 4). Statistical significance was calculated *via* ordinary one-way ANOVA with a Tukey's test. ns, no significance.

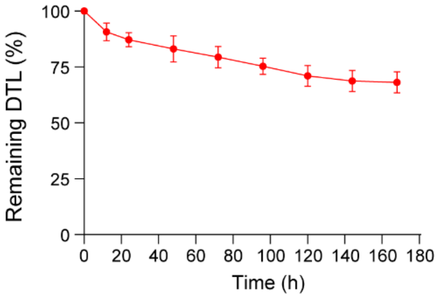

Figure S10. The percentage of remaining DTL in the hydrogel scaffold was measured at the indicated time points. All data are expressed as mean ± S.D. (n = 3).

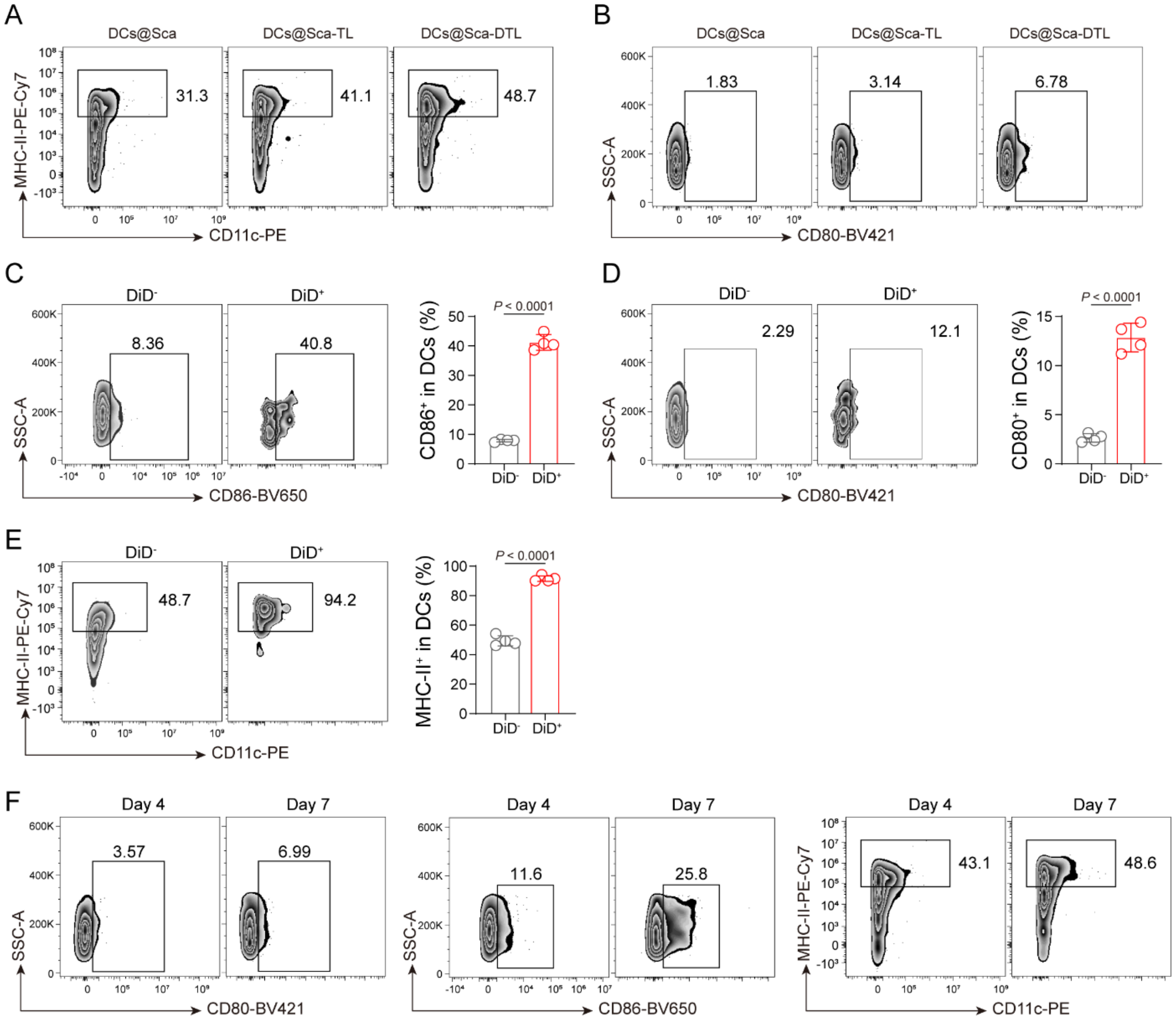

Figure S11. Flow cytometry analysis of DCs in lymph nodes. A,B) Representative flow cytometry analysis of MHC-II$^+$ DCs (A) and CD86$^+$ DCs (B) in draining lymph nodes after different treatments on day 7. C-E) Representative flow cytometry analysis and quantitation of the proportion of CD86$^+$ DCs (C), CD80$^+$ DCs (D), and MHC-II$^+$ DCs (E) in DiD$^+$ DCs and DiD$^-$ DCs in DCs@Sca-DTL on day 4. F) Representative flow cytometry analysis of CD80$^+$ DCs, CD86$^+$ DCs and MHC-II$^+$ DCs in the DCs of draining lymph node on day 4 and day 7 in DCs@Sca-DTL. All data are expressed as mean ± S.D. (n = 4). Statistical significance was calculated *via* ordinary one-way ANOVA with a Tukey's test.

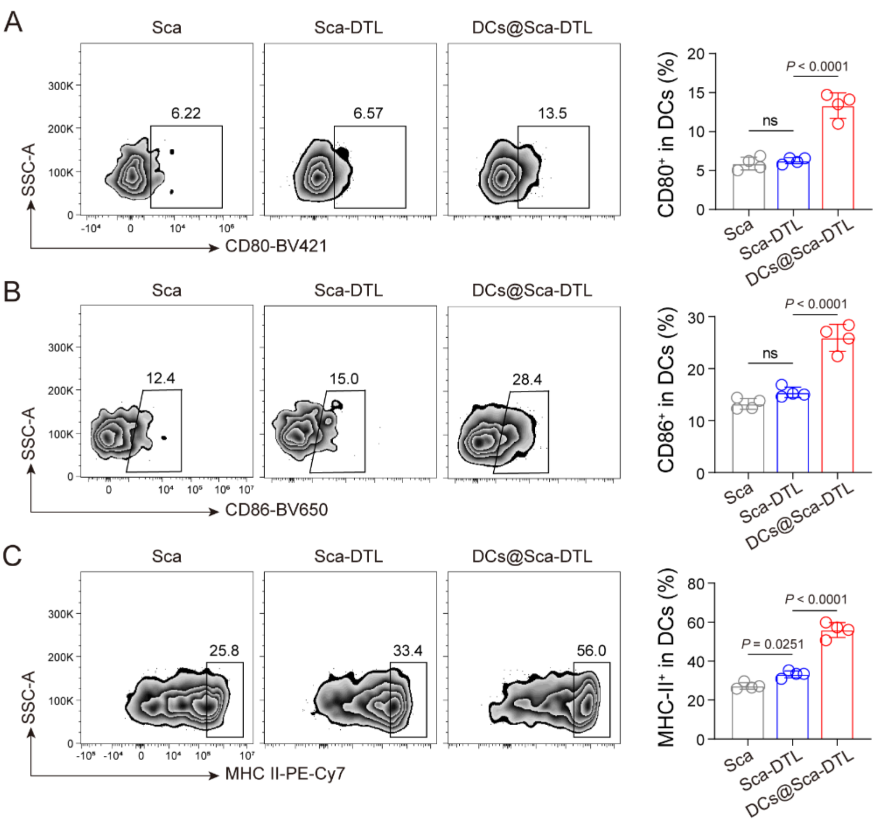

Figure S12. Flow cytometric analysis of DC maturation markers in dLNs. Representative flow cytometry analysis and quantitation of the proportion of $CD80^+$ (A), $CD86^+$ (B) and MHC-II^+ (C) DCs in the DCs of dLNs. All data are expressed as mean ± S.D. ($n = 4$). Statistical significance was calculated *via* ordinary one-way ANOVA with a Tukey's test.

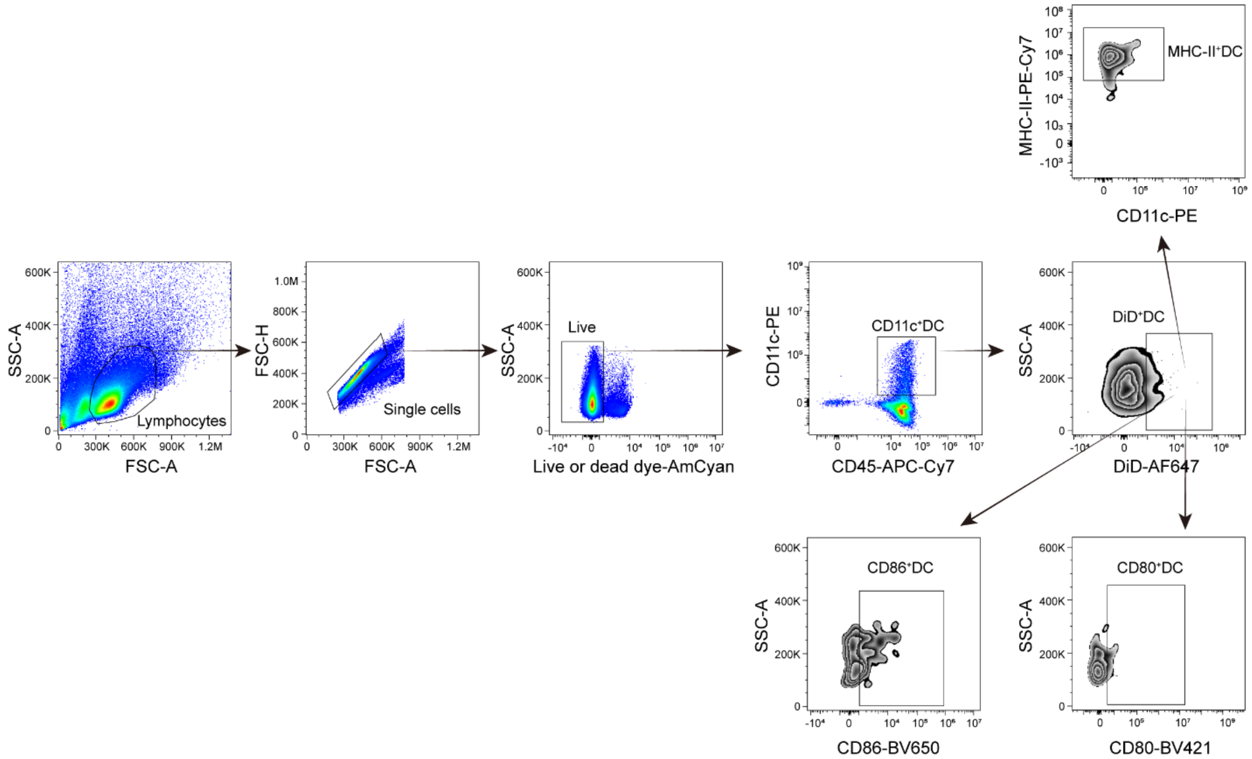

Figure S13. Flow cytometry gating strategy of lymph node cells in DCs@Sca-DTL group.

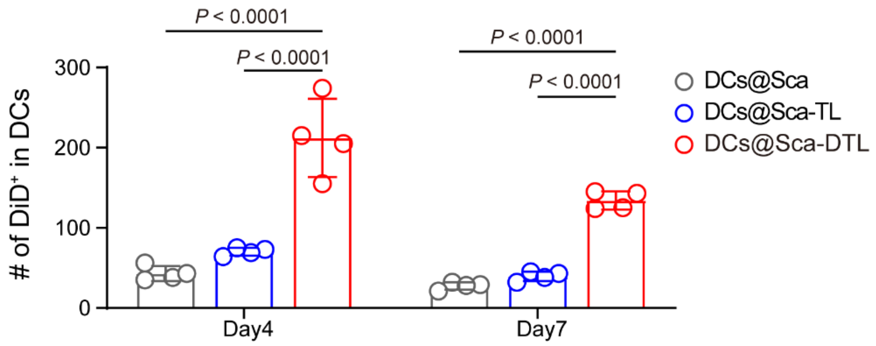

Figure S14. The number of migrated DCs in draining lymph nodes. '#' denotes number. All data are expressed as mean ± S.D. (n = 4). Statistical significance was calculated *via* ordinary one-way ANOVA with a Tukey's test.

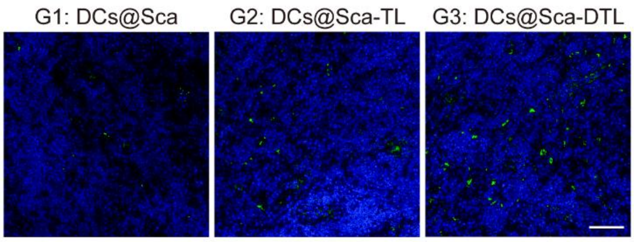

Figure S15. Immunofluorescence images of DiD^+ DCs in isolated dLNs. Scale bar, 40 μm.

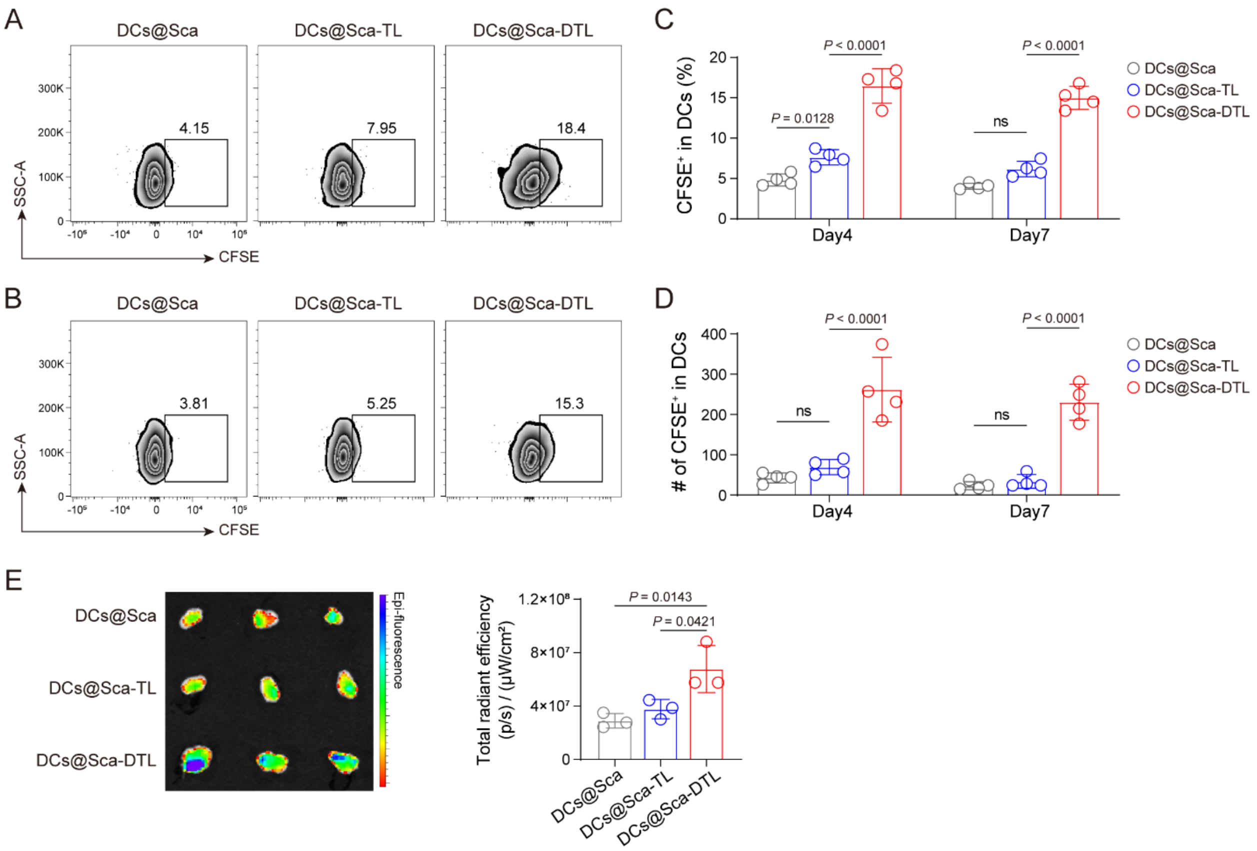

Figure S16. CFSE-labeled DCs in draining lymph nodes. Representative flow cytometry analysis of CFSE⁺ in DCs on day 4 (A) and 7 (B). C) Quantitation of the percentage of CFSE⁺ in DCs. D) The number of migrated DCs in draining lymph nodes. '#' denotes number. E) IVIS images and fluorescence intensities analysis of dLNs on day 7. All data are expressed as mean ± S.D. (C, D, *n* = 4; E, *n* = 3). Statistical significance was calculated *via* ordinary one-way ANOVA with a Tukey's test. ns, no significance.

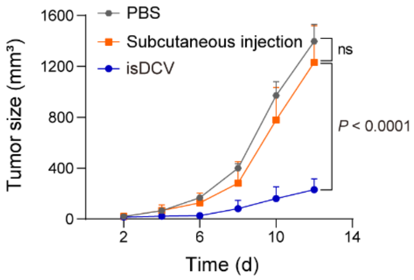

Figure S17. Average tumor growth curves of mice treated with PBS, subcutaneous injection of DC + DTL + GM-CSF + Flt3L, and isDCV. Identical cell numbers, antigen doses, and cytokine concentrations were used in the subcutaneous injection and isDCV groups. All data are expressed as mean ± S.D. (n = 5). Statistical significance was calculated *via* 2-way ANOVA with a Tukey's test.

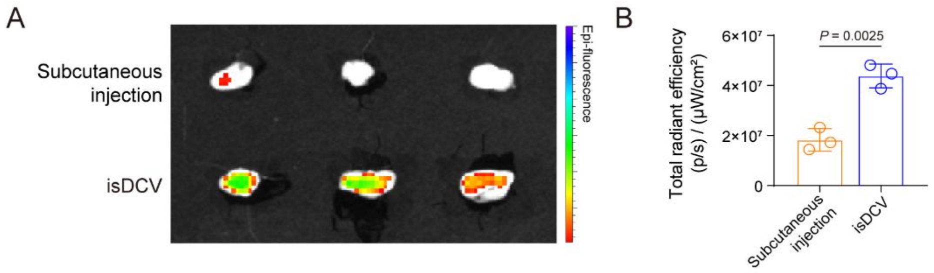

Figure S18. isDCV implantation enhances dendritic cell migration to draining lymph nodes. A) Representative IVIS images of CFSE-labeled DCs in dLNs at day 7 after treatment with either subcutaneous injection of CFSE-labeled DC suspension or implantation of CFSE-labeled DCs *via* isDCV. B) Quantification of DC migration based on CFSE fluorescence intensity in dLNs. All data are expressed as mean ± S.D. (n = 3). Statistical significance was calculated *via* unpaired two-tailed t-test.

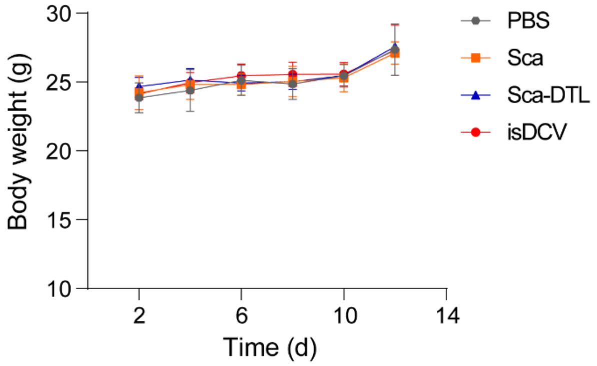

Figure S19. Average body weight curve of mice after different treatments. All data are expressed as mean ± S.D. (n = 5).

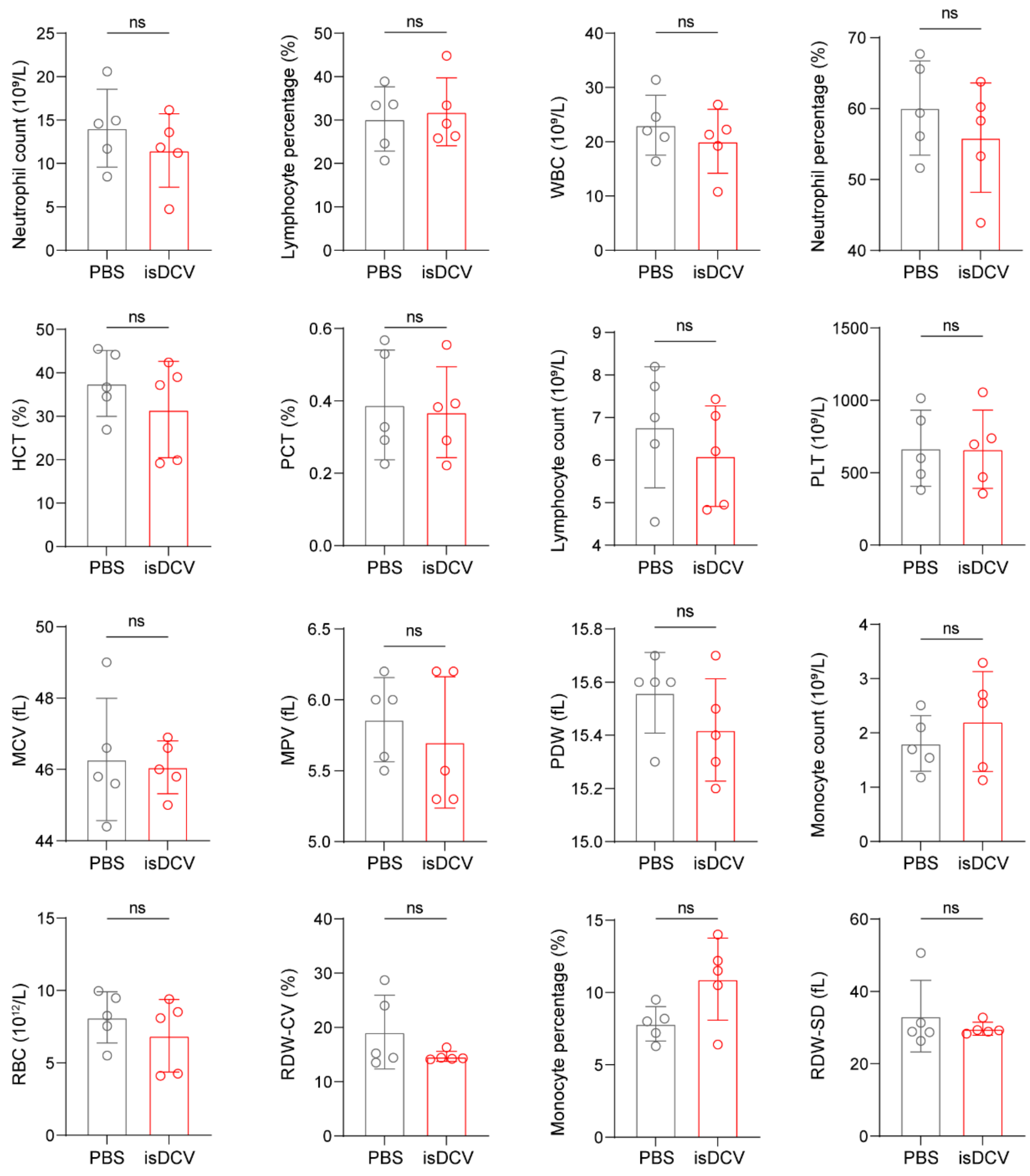

Figure S20. Blood analysis after treatments with PBS and isDCV. The relative expression analysis of neutrophil count, lymphocyte percentage, white blood cell (WBC), neutrophil percentage, hematocrit (HCT), platelet crit (PCT), lymphocyte count, blood platelet (PLT), mean corpuscular volume (MCV), mean platelet volume (MPV), platelet distribution width (PDW), monocyte count, red blood cell (RBC), red blood cell distribution width-coefficient of variation (RDW-CV), monocyte percentage, red blood cell distribution width-standard deviation (RDW-SD). All data are expressed as mean ± S.D. (n = 5). Statistical significance was calculated *via* unpaired two-tailed t-test. ns, no significance.

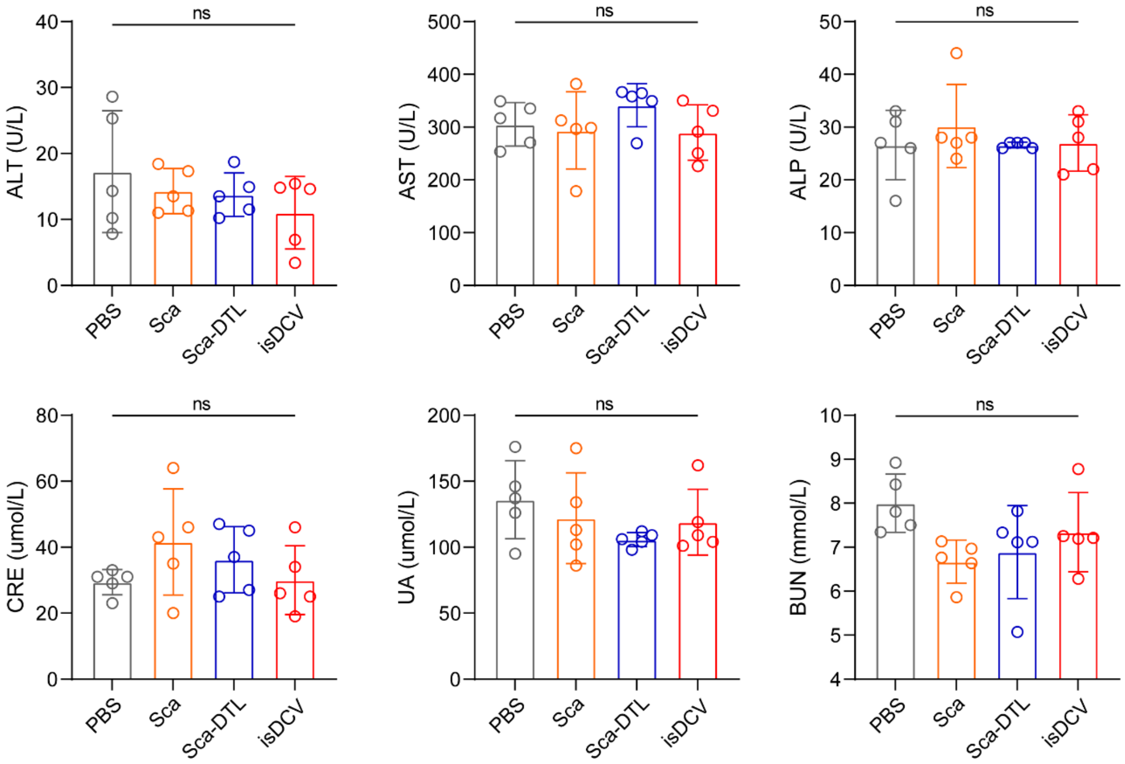

Figure S21. Blood biochemistry analysis after different treatments. The relative expression analysis of alanine transaminase (ALT), aspartate aminotransferase (AST), alkaline phosphatase (ALP), creatinine (CER), uric acid (UA) and blood urea nitrogen (BUN). All data were expressed as mean ± S.D. (n = 5). Statistical significance was calculated *via* ordinary one-way ANOVA with a Tukey's test. ns, no significance.

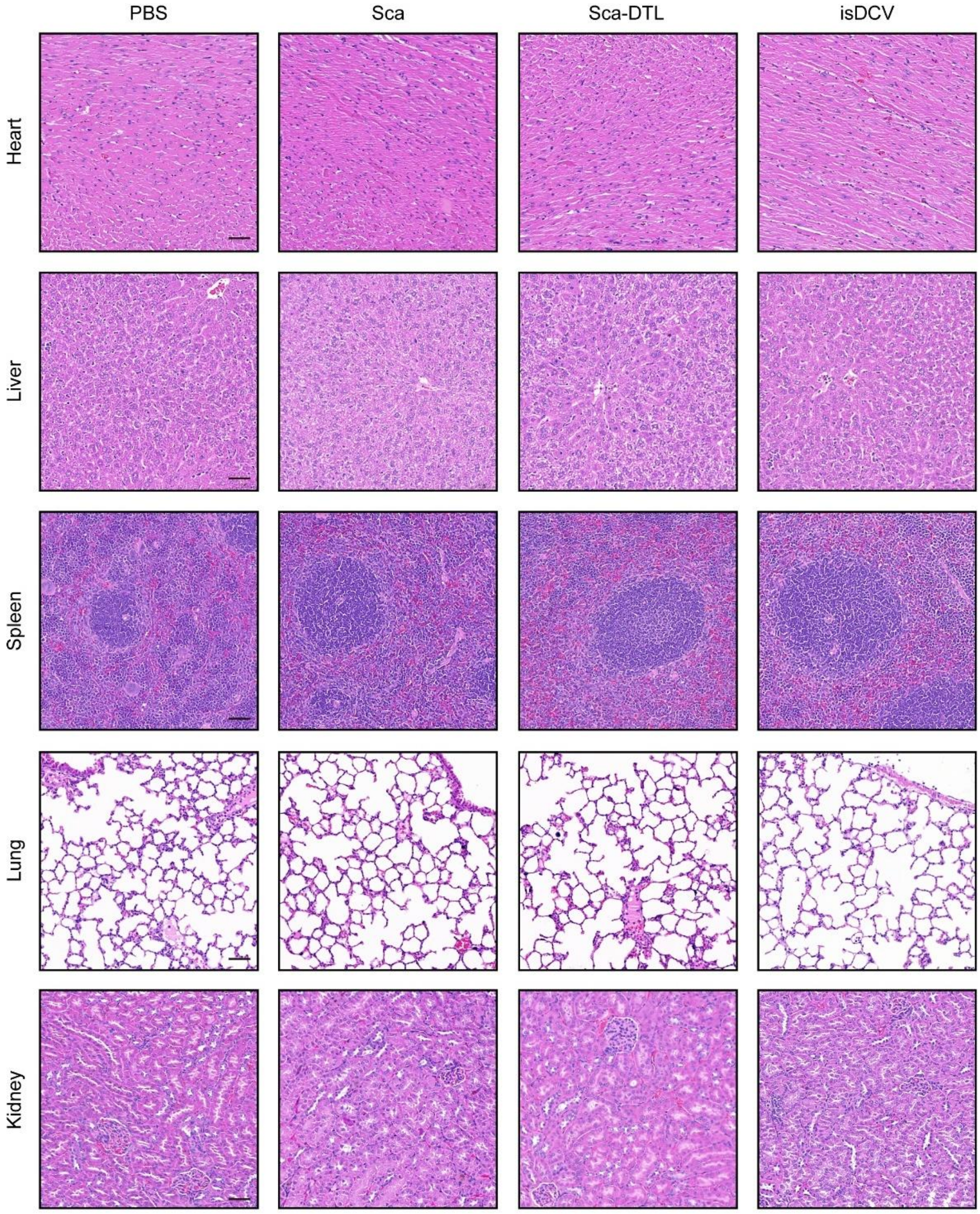

Figure S22. H&E images of tissues after different treatments. The H&E images of heart, liver, spleen, lung and kidney tissues after different treatments. Scale bars, 50 μm.

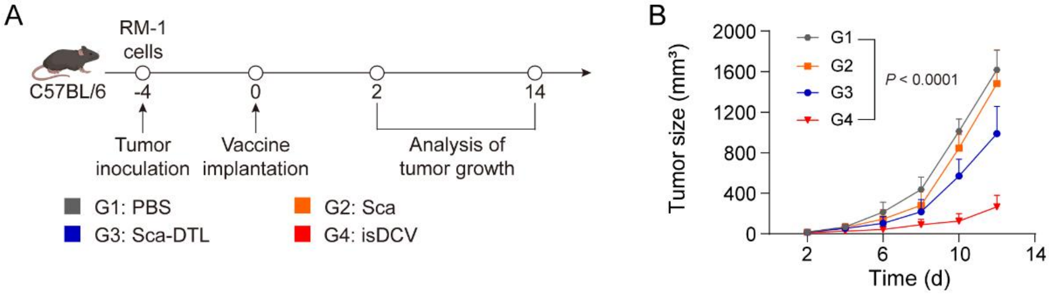

Figure S23. Antitumor efficacy of isDCV in a conventional non-surgical tumor vaccination model. A) Schematic illustration of the experimental design. RM-1 tumor cells were subcutaneously inoculated into C57BL/6 mice, followed by implantation of different vaccine formulations at sites spatially separated from the tumor bed. B) Tumor growth curves of RM-1 tumors after treatment. All data are expressed as mean ± S.D. (n = 5). Statistical significance was calculated *via* 2-way ANOVA with a Tukey's test.

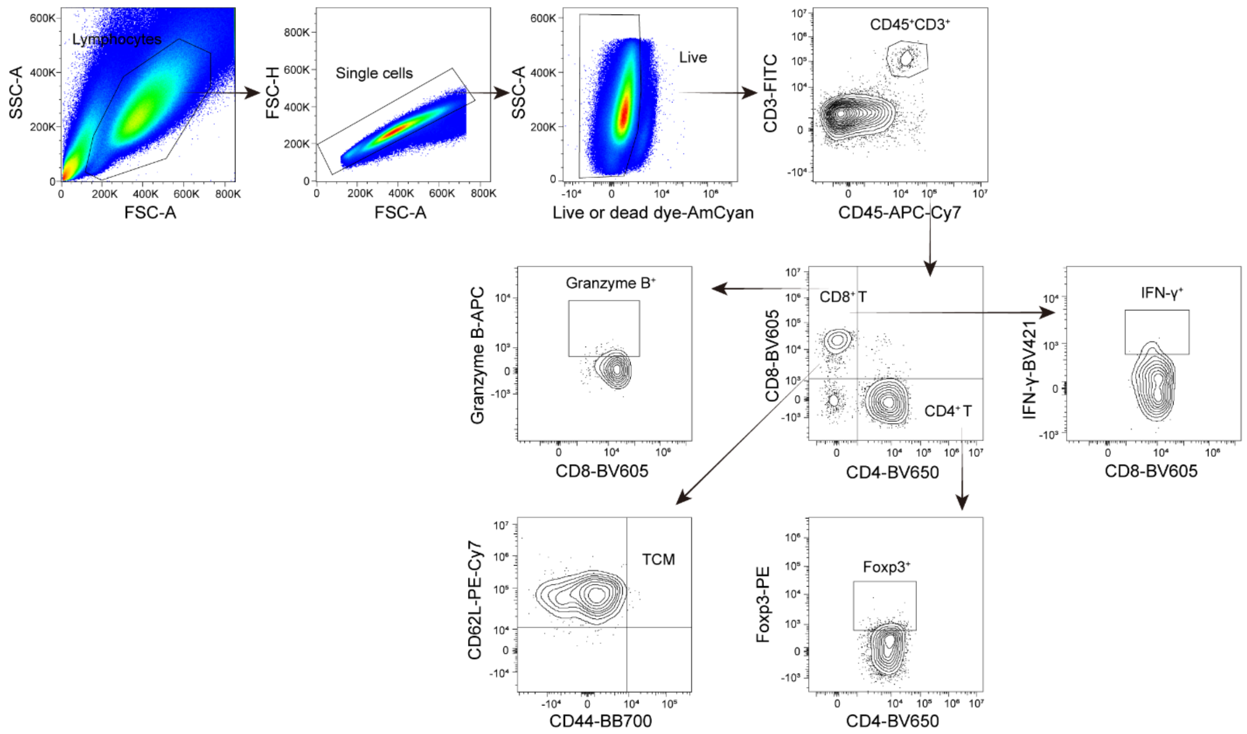

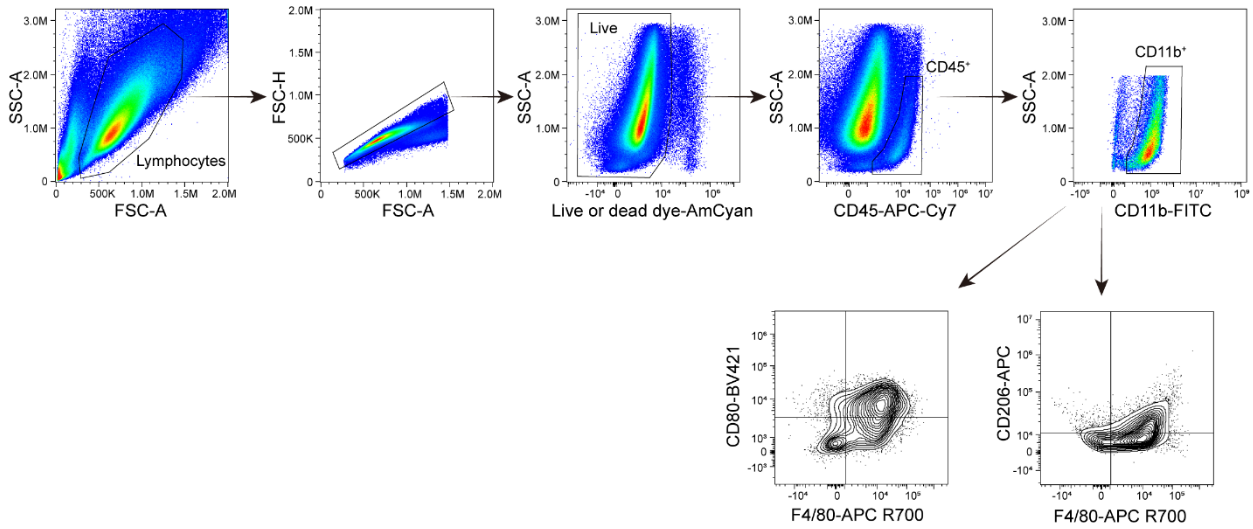

Figure S24. Flow cytometry gating strategy of tumor tissue. TCM: Central memory T cell.

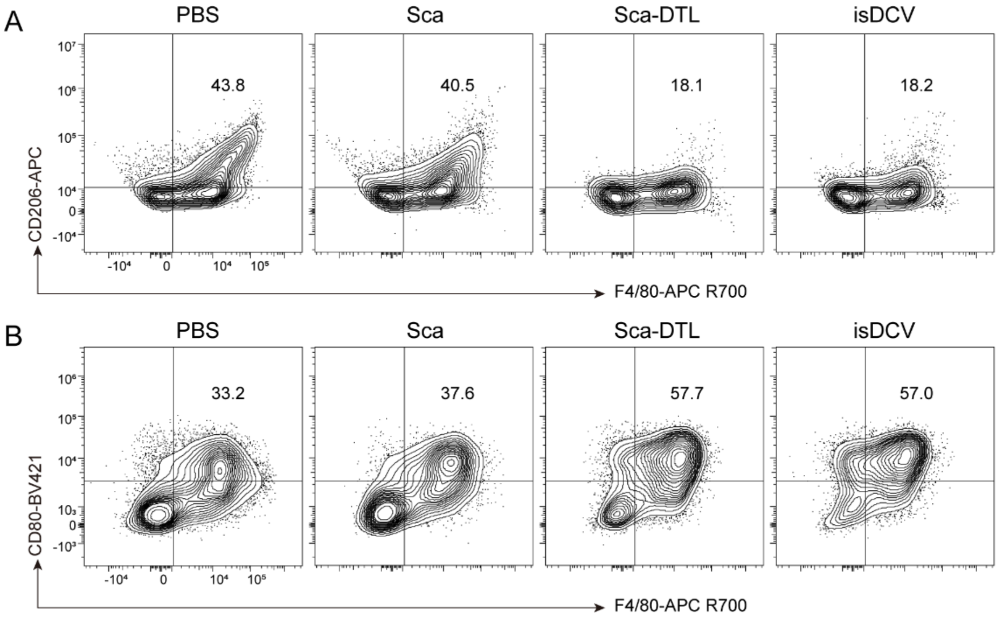

Figure S25. Flow cytometry analysis of recurrence tumor after different treatments. A) Representative flow cytometry analysis of M2-like macrophages ($CD206^+$) gating on $F4/80^+CD11b^+CD45^+$ cells within the tumor microenvironment. B) Representative flow cytometry analysis of M1-like macrophages ($CD80^+$) gating on $F4/80^+CD11b^+CD45^+$ cells within the tumor microenvironment.

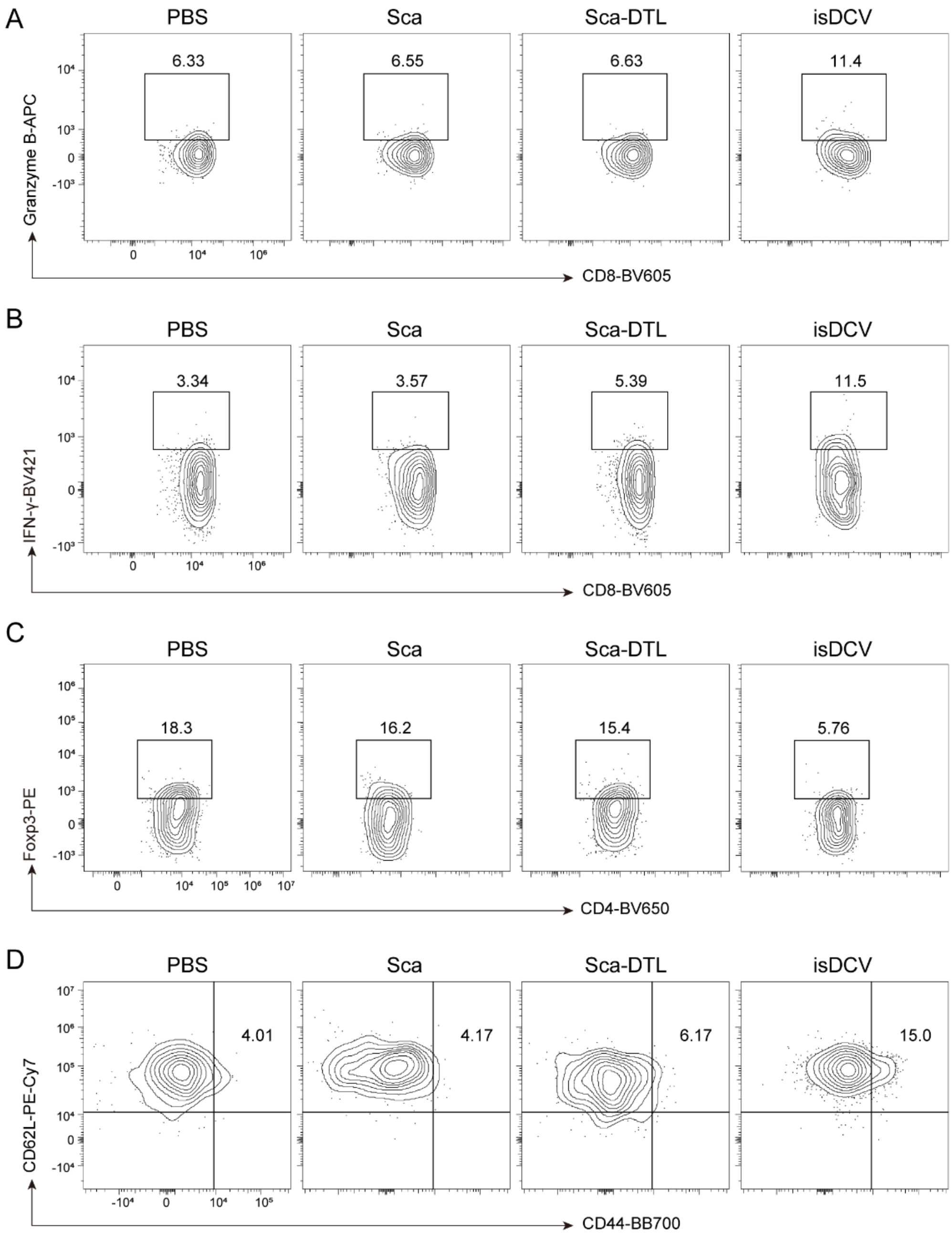

Figure S26. Representative flow cytometry analysis of Granzyme B⁺ in CD8⁺ T cells (A), IFN-γ⁺ in CD8⁺ T cells (B), Foxp3⁺ in CD4⁺ T cells (C), and CD44⁺CD62L⁺ in CD8⁺ T cells (D) in recurrence tumor after different treatments.

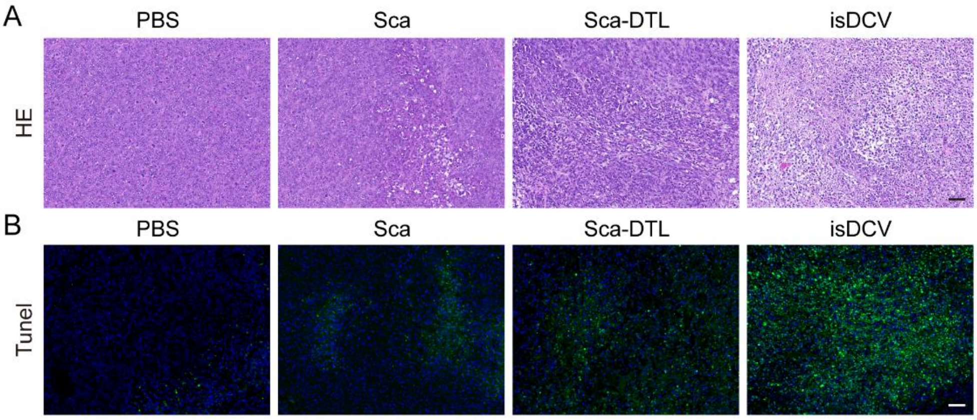

Figure S27. H&E and immunofluorescence images of tumor tissues after different treatments. A) H&E images of tumor tissues after different treatments. Scale bar, 50μm. B) Immunofluorescence images of tumor cell apoptosis after different treatments. Scale bar, 50 μm.

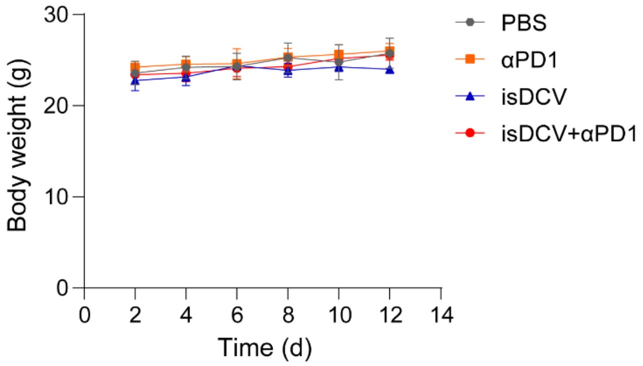

Figure S28. Average body weight curve of mice after different treatments. All data are expressed as mean ± S.D. (n = 5).

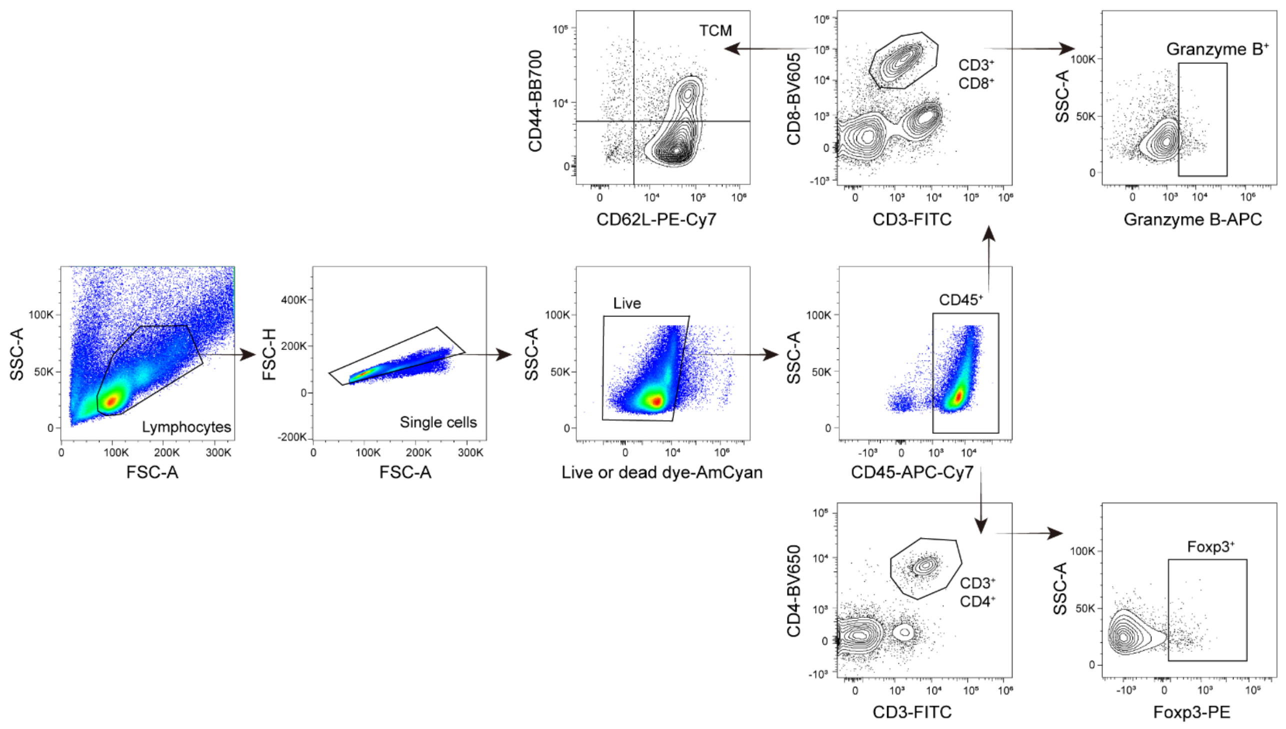

Figure S29. Flow cytometry gating strategy of spleen. TCM: Central memory T cell.

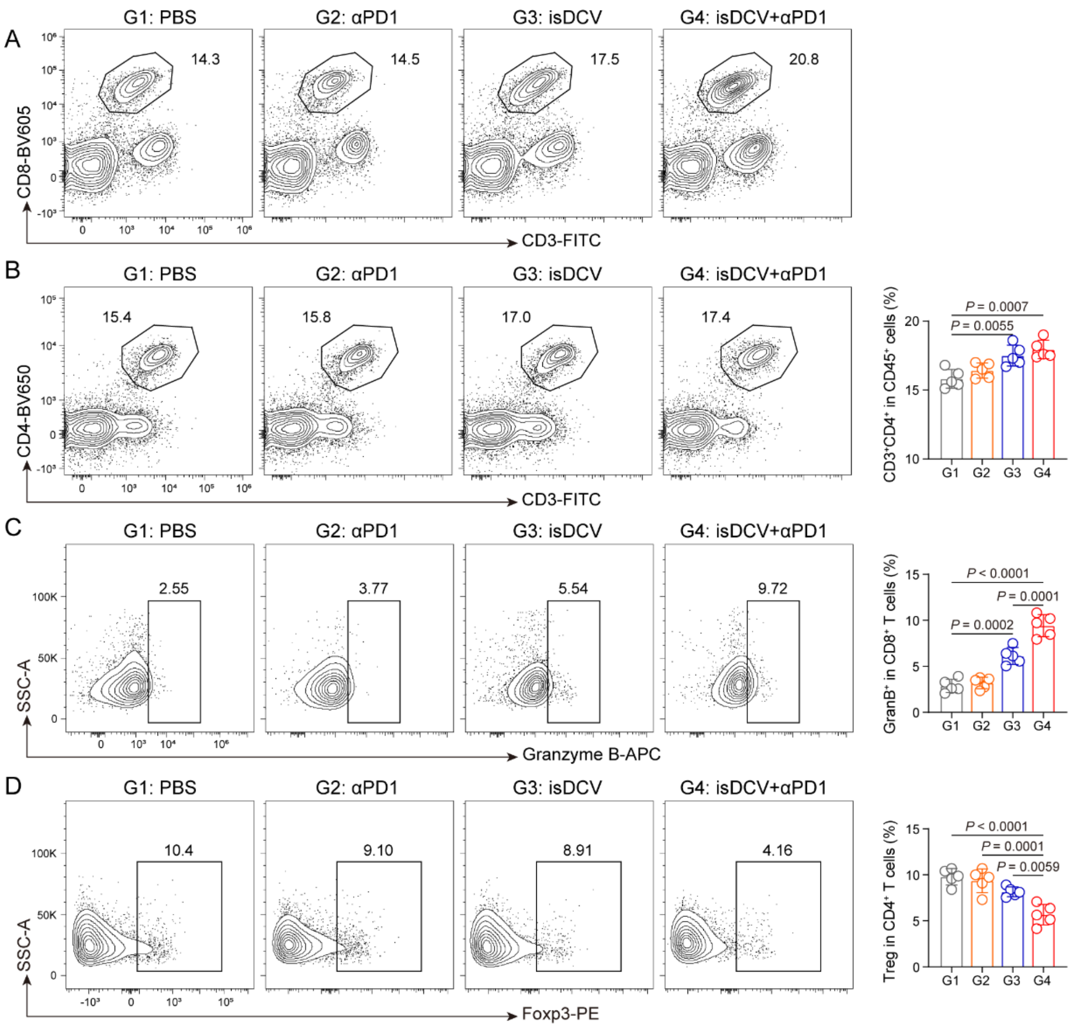

Figure S30. Flow cytometry analysis of draining lymph node and spleen after different treatments. A) Representative flow cytometry analysis of $CD3^+CD8^+$ T cells in $CD45^+$ cells. B) Representative flow cytometry analysis and quantitation of the proportion of $CD3^+CD4^+$ T cells in $CD45^+$ cells. C,D) Representative flow cytometry analysis and quantitation of the proportion of Granzyme B^+ in $CD8^+$ T cells (C), and $Foxp3^+$ in $CD4^+$ T cells (D). All data are expressed as mean ± S.D. (n = 5). Statistical significance was calculated *via* ordinary one-way ANOVA with a Tukey's test.

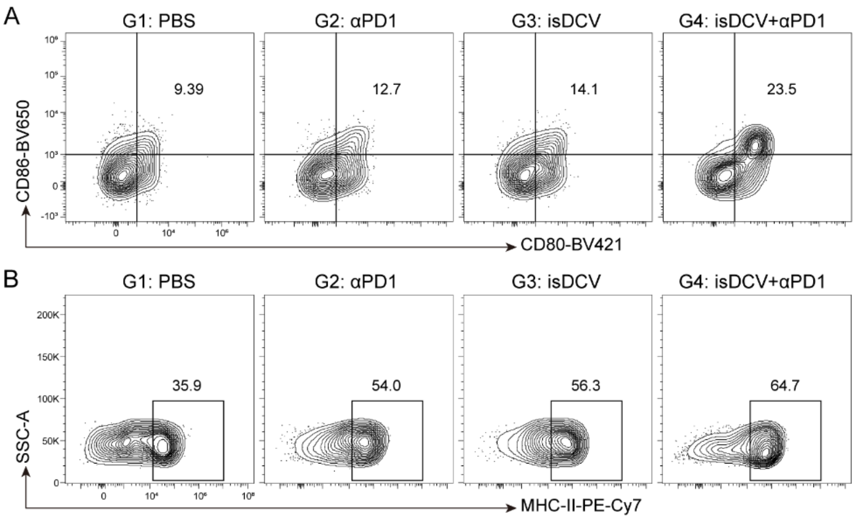

Figure S31. Representative flow cytometry analysis of DC maturity in DCs (A), and MHC-II⁺ in DCs (B) in draining lymph nodes after different treatments.

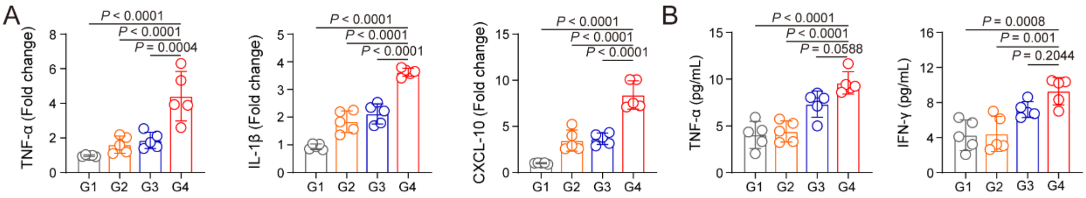

Figure S32. A) RT-qPCR showing up-regulated TNF-α, IL-1β and CXCL-10 in the recurrence tumor. B) Concentration of TNF-*α* and IFN-γ in the serum of mice after different treatments. All data are expressed as mean ± S.D. (n = 5). Statistical significance was calculated *via* ordinary one-way ANOVA with a Tukey's test.

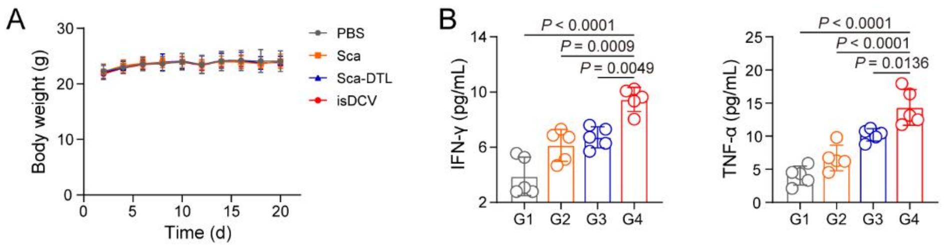

Figure S33. A) Average body weight curve of mice after different treatments. B) Concentration of TNF-α and IFN-γ in the serum of mice after different treatments. All data are expressed as mean ± S.D. (n = 5). Statistical significance was calculated *via* ordinary one-way ANOVA with a Tukey's test.

Supporting Table S1. The parameters of 3D printing.

Image	Exp-t	Intst	P-d	Dt1	P-u	Dt2	Scra	Rol
1	20	30	3	0	2.6	20	0	0
2	18	30	3	0	2.6	20	0	0
4	16	30	3	0	2.6	20	0	0

Image: Quantity of images in each stage;

Exp-t: UV exposure time;

Intst: UV intensity;

P-d: Distance for platform to go down;

Dt1: Delay time for platform to stay after going down;

P-u: Distance for platform to go up;

Dt2: Delay time for platform to stay after going up;

Scra: Frequency of scraper movement;

Rol: Frequency of Roller movement.

Supporting Table S2. The primers for qRT-PCR analysis of samples from mice.

genes	Forward primer (5'-3')	Reverse primer (5'-3')
Actin (beta)	GGCTGTATTCCCCTCCATCG	CCAGTTGGTAACAATGCCATGT
Zbtb46	CTTTCAGCAGCCGAGACTCA	GGCCCAAGTAGCTGGTTTCT
Irf4	GCCAGCCCAGGTTCATAACTA	CAGGTGGGGCACAAGCATAA
Irf8	CCGCCTATGACACACACCAT	CCATCCGGCCCATACAACTT
Ccr7	GAAACCCAGGAAAAACGTGCT	ACTCGTACAGGGTGTAGTCCA
Adgre4	TGGCCAACTACAGCAACTCA	AGCAGTGGTTGTGTGTTCCA
Vcam1	AAGGGACGATTCCGGCATTT	TCGGGCACATTTCCACAAGT
H2-Eb1	CTGTCACGGTCGAGTGGAAA	CCTGTTGGCTGAAGTCCAGA
IFN-γ	ATGAACGCTACACACTGCATC	CCATCCTTTTGCCAGTTCCTC
TNF-α	GGTGCCTATGTCTCAGCCTCTT	GCCATAGAACTGATGAGAGGGAG
IL-1β	ATGCCACCTTTTGACAGTGATG	TGTGCTGCTGCGAGATTTGA
CXCL-10	ATCATCCCTGCGAGCCTATCCT	GACCTTTTTTGGCTAAACGCTTTC
